# Pancreatic cancer cells render tumor-associated macrophages metabolically reprogrammed by a GARP and DNA methylation-mediated mechanism

**DOI:** 10.1038/s41392-021-00769-z

**Published:** 2021-10-29

**Authors:** Mengwen Zhang, Xingyi Pan, Kenji Fujiwara, Noelle Jurcak, Stephen Muth, Jiaojiao Zhou, Qian Xiao, Anqi Li, Xu Che, Zihai Li, Lei Zheng

**Affiliations:** 1 Department of Oncology, Johns Hopkins University School of Medicine, Baltimore, MD 21287 USA; 2 The Sidney Kimmel Cancer Center, Johns Hopkins University School of Medicine, Baltimore, MD 21287 USA; 3 The Pancreatic Cancer Precision Medicine Center of Excellence Program, Johns Hopkins University School of Medicine, Baltimore, MD 21287 USA; 4 The Cellular and Molecular Medicine Graduate Program, Johns Hopkins University School of Medicine, Baltimore, MD 21287 USA; 5 Pelotonia Institute for Immune-Oncology, The Ohio State University Comprehensive Cancer Center, Columbus, OH 43210 USA; 6 Department of Surgery, Johns Hopkins University School of Medicine, Baltimore, MD 21287 USA; 7 Present Address: The Second Affiliated Hospital, Zhejiang University School of Medicine, Hangzhou, China; 8 Present Address: Department of Surgery, Sada Hospital, Fukuoka, Japan; 9 Present Address: Cancer Hospital, Chinese Academy of Medical Sciences, Shenzhen, China

**Keywords:** Cancer microenvironment, Metastasis

## Abstract

How tumor-associated macrophages transit from a predominant antitumor M1-like phenotype to a protumoral M2-like phenotype during the development of pancreatic ductal adenocarcinoma (PDA) remains to be elucidated. We thus conducted a study by employing a PDA-macrophage co-culture system, an “orthotopic” PDA syngeneic mouse model, and human PDA specimens, together with macrophages derived from GARP knockout mice and multiple analytic tools including whole-genome RNA sequencing, DNA methylation arrays, multiplex immunohistochemistry, metabolism measurement, and invasion/metastasis assessment. Our study showed that PDA tumor cells, through direct cell–cell contact, induce DNA methylation and downregulation of a panel of glucose metabolism and OXPHOS genes selectively in M1-like macrophages, leading to a suppressed glucose metabolic status in M1-like but not in M2-like macrophages. Following the interaction with PDA tumor cells, M1-like macrophages are reprogrammed phenotypically to M2-like macrophages. The interaction between M1-like macrophages and PDA cells is mediated by GARP and integrin αV/β8, respectively. Blocking either GARP or integrin would suppress tumor-induced DNA methylation in *Nqo-1* gene and the reprogramming of M1-like macrophages. Glucose-response genes such as *Il-10* are subsequently activated in tumor-educated M1-like macrophages. Partly through *Il-10* and its receptor *Il-10R* on tumor cells, M1-like macrophages functionally acquire a pro-cancerous capability. Both exogenous M1-like and M2-like macrophages promote metastasis in a mouse model of PDA while such a role of M1-like macrophages is dependent on DNA methylation. Our results suggest that PDA cells are able to reprogram M1-like macrophages metabolically and functionally through a GARP-dependent and DNA methylation-mediated mechanism to adopt a pro-cancerous fate.

## Introduction

Pancreatic ductal adenocarcinoma (PDA) is one of the most stroma-rich cancers. Accumulated evidence suggests that the stroma in the tumor microenvironment (TME) plays a critical role in PDA progression and distant metastasis.^1,2^ The stroma is composed of fibroblast cells, immune cells, extracellular matrix, and many others. The naive stroma elements of the pancreas appear to be tumor suppressive as genetically depleting stroma elements promote the development and metastasis of PDA in the mouse model.^3,4^ However, cancer-associated fibroblasts (CAFs) in the tumor-reprogrammed stroma of PDA acquire pro-cancerous capabilities.^5^ Macrophages form another major stromal component of PDA. Bone marrow-derived macrophages (BMDMs), which give rise to the recruited macrophages in the tissue compartments including neoplasms, are polarized to distinct phenotypic and functional subsets including M1 macrophages and several types of M2 macrophages in response to various activation stimuli.^6^ Generally speaking, M1 macrophages have an antitumor capability, whereas M2 macrophages have a protumoral capability.^7^ Macrophages show a high level of functional plasticity at different tissue compartments and at different stages of tumor progression. The dichotomy classification of macrophages into M1 and M2 macrophages may not be appropriate. In vivo, only M1 or M2-like macrophages may exist. It is anticipated that tumor-associated macrophages (TAMs) are comprised of both BMDMs and tissue-resident macrophages.^8^ TAMs in the invasive malignancies show higher expression of specific surface receptors such as the mannose receptors,^9^ consistent with a M2-like phenotype.

Macrophages exhibit heterogeneous phenotypes as PDAs progress from pre-malignant pancreatic intraepithelial neoplasia (PanINs) to invasive adenocarcinomas. Early-stage PanINs are predominantly infiltrated with M1-like macrophages whereas the M2-like subtype is the dominant phenotype of macrophages in late-stage PanINs and invasive PDA.^10,11^ Studies have suggested that a higher density of M2-like macrophages in primary PDA was associated with a more advanced stage, a higher incidence of metastases and shorter survival.^12^ How TAMs transit from a predominant M1-like phenotype to a M2-like phenotype during the PDA development would need to be elucidated.

It would be an oversimplification of their complex biological properties to view macrophages as two polar counterparts. Although M1-like and M2-like macrophages not only differ phenotypically in their cell-surface marker expression and cytokine/chemokine expression but also in their metabolic states,^13^ evidence has supported the phenotypic switch between M1-like and M2-like macrophages, particularly in their metabolic states.^14^ Metabolism is shifted toward glycolysis with increasing uptake of glucose in classically activated M1-like macrophages compared to alternatively activated M2-like macrophages. There is also a transition in the demand for oxidative phosphorylation (OXPHOS) during the activation of M2-like macrophages.^15^ The distinct metabolic profiles of macrophages are intimately linked to their phenotypic status and functions.^16^ OXPHOS is shown to plays a role in mediating antitumor immunity in TAMs.^17^ Therefore, it is conceived that TAMs can be metabolically reprogramed in the TME.^13^ This hypothesis is supported by a recently published study showing that macrophages are programmed by PDA cells through a paracrine mechanism, leading to the release of a spectrum of pyrimidine species including deoxycytidine, which inhibits the drug uptake and metabolism of gemcitabine and subsequently suppresses the treatment response of PDA to gemcitabine.^18^ Nevertheless, whether metabolic reprogramming of macrophages plays a role in PDA development and metastasis still lacks evidence. Moreover, why a M1-like macrophage predominant stroma fails to reject tumor cells at the site of PDA initiation and metastasis remains unknown.^10^ Epigenetic regulation such as DNA methylation can be dynamically modulated through DNA methylation enzymes and has been suggested to provide a dynamic and reversible modulation of stromal fibroblasts by our prior study.^5^ Therefore, it would be intriguing to investigate whether a tumor-induced DNA methylation mechanism plays a role in the metabolic reprogramming of M1-like macrophages. In the present study, we investigate a novel DNA methylation-dependent mechanism by which tumor cells reprogram TAMs phenotypically, metabolically, and functionally through direct contact.

## Results

### PDA tumor cells induce DNA methylation of the *NQO-1* and *ALDH1a3* genes in macrophages

**Fig. 1:**
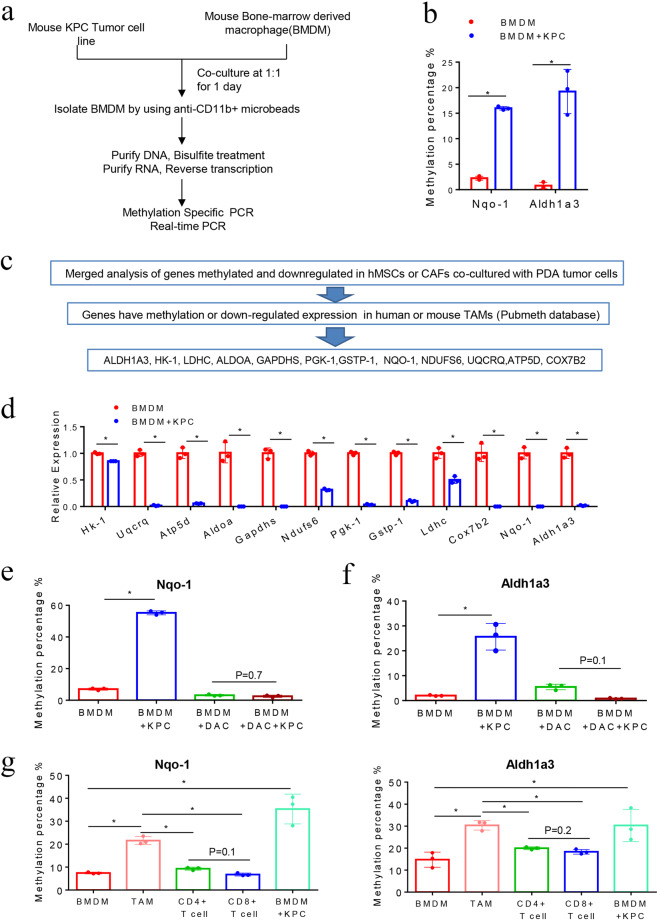
PDA cells reprogram macrophages in TME through DNA methylation. **a** Tumor and macrophage co-culture experimental schema. **b** *Nqo-1* and *Aldh1a3* methylation was examined by methylation-specific PCR (MSP) in mouse BMDMs after co-culturing with KPC PDA cells. *P < 0.05 (paired *t* test). **c** The schema of the candidate gene selection process. **d** Expression of the key genes in glucose metabolism and OXPHOS pathway in mouse BMDMs after co-culturing with KPC cells. The mRNA expression of these genes was measured by RT-PCR and *β-actin* was used for normalization. **e**, **f** *Nqo****-****1* and *Aldh1a3* methylation after pretreating BMDMs with DAC. *P < 0.05 (**d***,* **e**, **f**, Mann–Whitney *U* test)**. g** Methylation of *Nqo****-****1* and *Aldh1a3* in TAMs, CD4^+^ and CD8^+^ T cells from primary PDA and BMDMs (consider as M0 macrophages) of the same KPC mice, and BMDMs after co-culturing with KPC cells. *P < 0.05 (ANOVA). Data are means ± SEM from technical duplicates and representative of two experiments

When we were studying the interaction between the neoplastic cells and stromal fibroblasts in the PDAs, we found that neoplastic cells can induce DNA methylation at a whole-genome level in CAFs.^5^ Prior to our study, Shakya et al. had compared the gene expression profiling between DNA-demethylating agent-treated CAFs and untreated CAFs from the PDA tumors of a genetically engineered mouse model of PDA, the KPC-Brca1 mouse model,^19^ and found that *Aldh1a3* in the glucose metabolism pathway and *Nqo-1* in the OXPHOS pathway was regulated by DNA methylation. Remarkably, we found that most of the key genes in the *Aldh1*-associated metabolism pathway and *Nqo-1*-associated OXPHOS pathway have increased methylation levels and also were downregulated in CAFs following a 24-hour co-culture with neoplastic cells. These include 13 *Aldh1a3*-related genes in the glucose metabolism pathway, 7 *Aldh1a3*-related genes in other metabolism pathways, and 15 *Nqo-1*-related genes in the OXPHOS pathway (Supplementary Table 1). We hypothesized a similar interaction occurs between neoplastic cells and macrophages in the PDAs and, as a result of this interaction, tumor-induced DNA methylation also occurs in the macrophages of PDAs. We chose *Aldh1a3* and *Nqo-1*, whose methylation sequences were the best defined, to be examined for tumor-induced methylation in macrophages by MSP. For macrophages, we used BMDMs, which were thought to be the origin of TAMs. Murine BMDMs have a low level of *Aldh1a3* and *Nqo-1* methylation as expected. We co-cultured mouse BMDMs with mouse KPC PDA tumor cells for 24 h, followed by isolation of the tumor-educated macrophages by anti-CD11b magnetic beads (Fig. 1a). *Aldh1a3* and *Nqo-1* methylation were both induced in mouse BMDMs after co-culturing (Fig. 1b). Supporting this hypothesis, among the 35 methylated and downregulated, metabolism-related genes identified in CAFs co-cultured with neoplastic cells in our previously published study,^5^ promoters of 12 genes were reported in the Pubmeth Database to be regulated by DNA methylation in TAMs. These 12 genes include *Aldh1a3*, *Hk-1, Aldoa, Gapdhs, Pgk-1, Ldhc* in the glycolysis pathway, *Gstp-1* in the hepatic gluconeogenesis pathway, and *Nqo-1*, *Ndufs6*, *Uqcrq*, *Atp-5d*, *Cox7b2* in the OXPHOS pathway (Fig. 1c). Subsequently, quantitative RT-PCR confirmed that all the key genes in the glucose metabolism and OXPHOS pathways were downregulated in mouse BMDMs after co-culturing with mouse PDA cells (Fig. 1d). By contrast, *Nqo-1* and *Aldh1a3* hypermethylation were no longer induced in mouse BMDMs after pretreated with DAC, a DNA methyltransferases (DNMTs) inhibitor, before co-culturing with PDA cells (Fig. 1e, f). These results suggest that PDA cells induce DNA methylation in the *Nqo-1* and *Aldh1a3* genes in BMDMs. We then compared the methylation levels of *Nqo-1* and *Aldh1a3* in tumor infiltrated immune cells including TAMs, CD4^+^T cells, and CD8^+^T cells to BMDMs from the same KPC mouse. The methylation level of *Nqo-1* and *Aldh1a3* in TAMs, but not in CD4^+^ and CD8^+^ T cells, were significantly higher than that in BMDMs and similar to that in BMDMs after co-culturing with PDA cells (Fig. 1g). We found that the baseline methylation level of *Nqo-1* and *Aldh1a3* in BMDMs from KPC mice appears to be higher than that in normal mice (Fig. 1b, g). It is possible that a small number of circulating PDA tumor cells have infiltrated into the bone marrow and induced a modest elevation of the *Nqo-1* and *Aldh1a3* methylation in BMDMs in KPC mice. Nevertheless, we also cannot exclude the possibility that other factors directly or indirectly related to tumor development may have elevated the baseline level of *Nqo-1* and *Aldh1a3* in BMDMs. Taken together, the above results suggest that the methylation of *Nqo-1* and *Aldh1a3* or possibly more genes in the glucose metabolism and OXPHOS pathways is induced and their gene expression is suppressed in BMDMs after co-culturing with PDA cells as a result of tumor-induced DNA methylation.

### The DNA methylation of these metabolism genes in macrophages is induced by direct interaction with pancreatic tumor cells

**Fig. 2:**
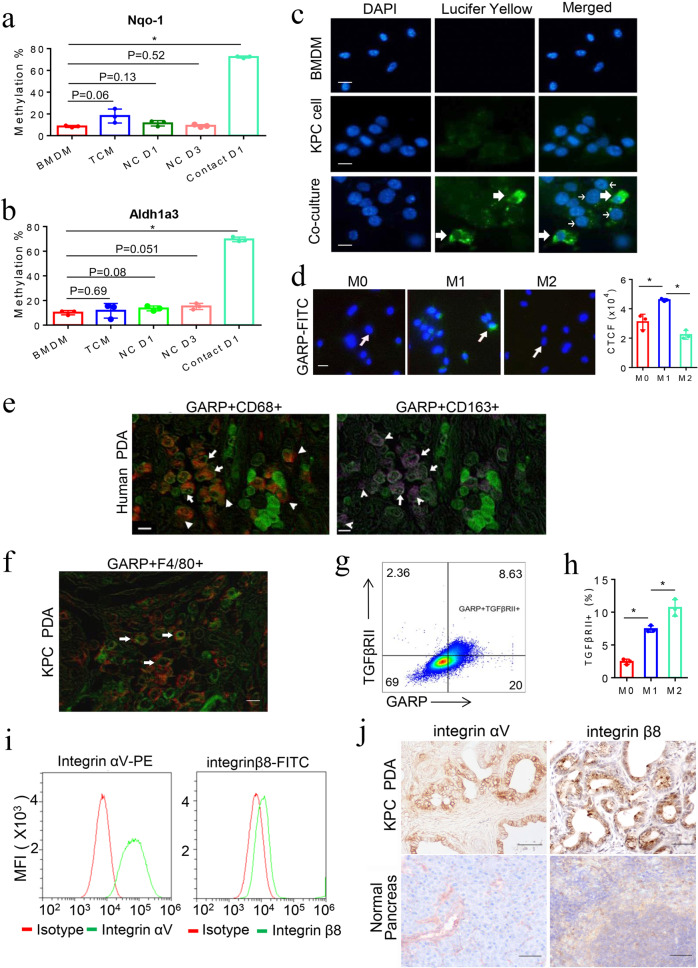
DNA methylation of the metabolism genes in macrophages is induced by direct interaction with PDA cells through GARP/TGF-βRII-integrin αV/β8. **a**, **b** *Nqo-1* and *Aldh1a3* methylation in mouse BMDMs in a transwell system separated from KPC cells by an 8-μm or 1-μm pore membrane that, respectively, allows or not allows tumor cells to migrate through and direct contract with macrophages and in BMDMs cultured with TCM. *Nqo-1* and *Aldh1a3* methylation quantified as described in Supplementary Methods. *P < 0.05 (Mann–Whitney *U* test). **c** Lucifer Yellow labeled-KPC cells were co-cultured with unlabeled BMDMs. Thick arrows indicate macrophages that contain Lucifer Yellow spread from KPC cells (thin arrow) around them. Scale bar: 20 μm. **d** GARP expression on M0, M1-like, and M2-like macrophages measured by immunofluorescent staining with FITC-conjugated anti-GARP antibody. Arrow indicates macrophages that have the highest fluorescence within each image. Scale bar: 20 μm. *P < 0.05 (ANOVA). Histogram (right panel) shows quantification of fluorescence intensity. **e** Multiplex immunohistochemistry (IHC) was performed on a single slide of human PDA tissues for GARP (in green), CD68 (in red) and CD163 (in purple). A representative among 20 human PDAs tested is shown. Arrows (both panels) indicate GARP-expressing CD68^+^CD163^+^ (M2-like) macrophages^;^ and arrowheads (left panel) indicate GARP-expressing CD68^+^CD163^-^ (M1-like) macrophages^.^ Notched arrowheads (right panel) indicate CD68^+^CD163^+^ (M2-like) macrophages with little GARP expression. Scale bar: 50 μm. **f** Multiplex IHC staining of GARP (in green) on F4/80^+^ (in red) macrophages in PDAs from KPC mice. Scale bar: 50 μm. **g** TGF-βRII and GARP on cell surface of M0, M1-like, and M2-like macrophages co-stained and analyzed by flow cytometry. **h** Quantification of the percentages of TGF-βRII on cell surface of M0, M1-like, and M2-like macrophages by flow cytometry. *P < 0.05 (ANOVA). **i** Integrin subunits ɑV and β8 cell-surface expression was measured by flow cytometry. **j** IHC staining of PDA and normal pancreas tissues from KPC mice with anti-integrin ɑV and β8 antibodies. Scale bar: 100 μm. Data were from technical triplicates and representative of two experiments

Previously, we identified a direct cell-to-cell interaction between PDA cells and CAFs.^5^ We wondered whether the methylation of the metabolism genes in mouse BMDMs was mediated by a similar cell-to-cell contact between tumor cells and macrophages. To this end, we examined whether the methylation of *Nqo-1* and *Aldh1a3* in BMDMs can be induced by a tumor-conditioned medium (TCM). However, even ten times (10×) concentrated TCM was not able to induce the methylation of *Nqo-1* and *Aldh1a3*. When BMDMs and mouse KPC PDA cells were co-cultured in a transwell system separated by a 1-μm pore semitransparent membrane (non-contact co-culture), inhibiting migration of PDA cells from the upper chamber to the lower chamber where macrophages were seeded, methylation of *Nqo-1* and *Aldh1a3* in mouse BMDMs was not induced after 1 or 3 days of co-culture. By contrast, when BMDMs and PDA cells were co-cultured in a transwell system separated by an 8-μm pore membrane (contact co-culture), allowing migration of PDA cells toward macrophages, methylation of both *Nqo-1* and *Aldh1a3* was observed after 1 day of co-culture (Fig. 2a, b). We further demonstrated that Lucifer Yellow can spread to macrophages from pre-stained PDA cells after cell-to-cell interaction (Fig. 2c), supporting the direct contact between PDA cells and macrophages. We also conducted the immunofluorescent staining of co-cultured PDA cells and macrophages, respectively, with their lineage markers (Pan-CK and F4/80). In addition, PDA cells can be distinguished from macrophages according to the size of the nucleus. PDA cells and macrophages migrated together through the transwell, further supporting the direct contact between them (Supplementary Fig. 1a, b).

### Latent TGF-β binding partner GARP is selectively expressed on M1-like macrophages

**Fig. 3:**
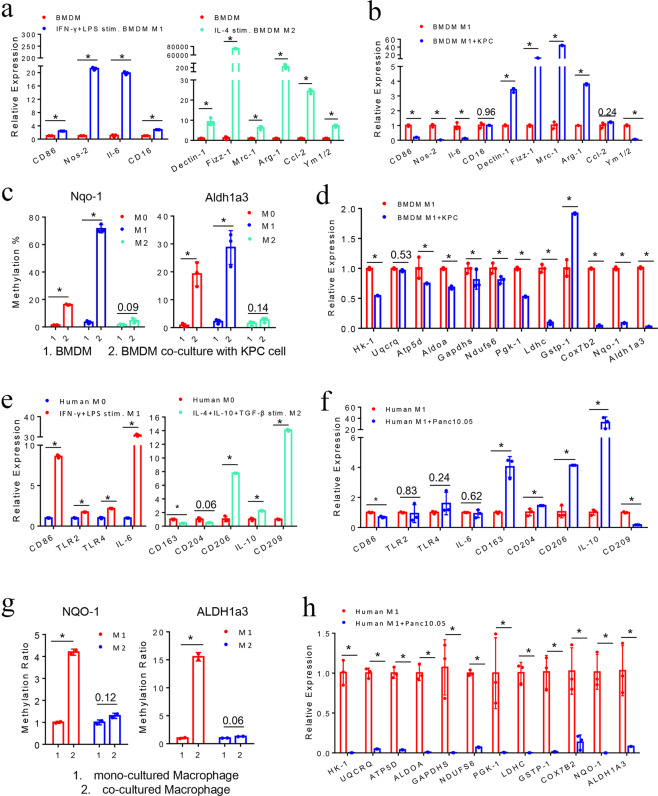
M1-like, but not M2-like macrophages are selectively reprogrammed by tumor-induced methylation. **a** Phenotypic characterization of mouse BMDMs after IFN-γ/LPS-induced M1 polarization and IL-4-induced M2 polarization. mRNA expression of M1 and M2 marker genes measured by RT-PCR with *β-actin* used for normalization. **b** M1 and M2 marker gene expression in mouse M1-like macrophages after co-culturing with KPC cells. **c** *Aldh1a3* and *Nqo-1* methylation in mouse M0, M1-like, and M2-like macrophages upon co-culturing with KPC cells. **d** RT-PCR of key genes in glucose metabolism and OXPHOS pathways in mouse M1-like macrophages after co-culturing with KPC cells. **e** Phenotypic characterization of human macrophages after IFN-γ/LPS-induced M1 polarization and IL-4/IL-10/TGF-β-induced M2 polarization. **f** RT-PCR of M1 and M2 marker genes in human M1-like macrophages after co-culturing with Panc10.05 cells. **g** *ALDH1a3* and *NQO-1* methylation in human M1-like and M2-like macrophages upon co-culturing with Panc10.05 cells. *P < 0.05 (paired *t* test). **h** RT-PCR of key genes in glucose metabolism and OXPHOS pathways in human M1-like macrophages after co-culturing with Panc10.05 cells. Data are means ± SEM from technical duplicates. *P < 0.05 (All panels except **g** used Mann–Whitney *U* test)

Our next step was to identify the cell-surface mediators that mediate this direct interaction between PDA cells and macrophages. GARP, a type I transmembrane cell-surface docking receptor for latent TGF-β, can interact with integrin subunits αV/β8 expressed on tumor cells and plays an important role in establishing the immunosuppressive TME through modulating Treg functions.^20,21^ The primary role of GARP in Treg was to regulate the availability of membrane-bound latent TGF-β and modulate its activation.^20,21^ Yet its role in myeloid cells was less defined. However, it is known that TGF-β signaling activation in macrophages can lead to the polarization of macrophages to adopt a M2-like phenotype.^22^ In addition, it was previously demonstrated that the binding of integrin αV/β8 to GARP would lead to the release of latent TGF-β from GARP and subsequent activation of TGF-β.^23^ Thus, we hypothesized that the interaction between GARP/TGF-βRII and integrin αV/β8 could potentially serve as cell-surface mediators that confer tumor-macrophage interactions and subsequently activate TGF-β signaling that reprogram macrophages to adopt a M2-like phenotype. To test this hypothesis, we first examined GARP/TGF-βRII expression on macrophages and integrin αV/β8 expression on mouse KPC PDA cells. We used different cytokines to polarize BMDMs toward the M1-like or M2-like phenotype according to the literature.^24^ A marked increase in M1 markers including *CD16, CD86, Nos-2*, and *Il-6* expression was observed in macrophages polarized by LPS and IFN-γ. By contrast, a marked increase in M2 markers including *Dectin-1*, *Fizz-1*, *Mrc-1*, *Arg-1*, *Ccl-2*, and *Ym1/2* expression were observed in macrophages polarized by IL-4 (Fig. 3a, below). Using both immunofluorescent staining and cell-surface flow cytometry staining, we found that GARP was highly expressed on the membrane of M1-like macrophages, less on M0 macrophages (unpolarized macrophages), and the least on M2-like macrophages (Fig. 2d). Using multiplex immunohistochemistry (mIHC), we demonstrated that GARP was expressed on CD68^+^ macrophages and CD163^+^ macrophages in all human PDA tissues tested (Fig. 2e and Supplementary Fig. 1c). As anticipated, not all the GARP-expressing cells are macrophages as GARP is known to be expressed on other stromal cells including lymphocytes.^25^ GARP is expressed on essentially all the CD68^+^ macrophages, including many CD68^+^CD163^-^ M1-like macrophages. On another hand, some of CD68^+^CD163^+^ M2-like macrophages, which may represent those M1-like macrophages reprogrammed by PDA cells as below suggested, also express GARP. In addition, mIHC demonstrated the expression of GARP on F4/80^+^ macrophages in the spontaneously formed PDA tumors from the KPC mice (Fig. 2f and Supplementary Fig. 1d). Flow cytometry analysis showed that TGF-βRII was co-expressed with GARP on mouse M1-like macrophages (Fig. 2g) and revealed that cell-surface expression of TGF-βRII was higher in M2-like macrophages than in M1-like macrophages (Fig. 2h and Supplementary Fig. 1e), in consistency with the previous findings.^26^

The IHC analysis further demonstrated that integrin subunits αV/β8 were co-expressed on cultured PDA cells, as well as on primary PDA tumors from all KPC mice tested (Fig. 2i, j and Supplementary Fig. 1f). We thus examined the expression of integrin subunits αV/β8 in human PDAs by analyzing the TCGA data. Both integrin subunits αV and β8 exhibited a significantly higher mRNA expression in human PDAs than the normal pancreatic tissues (Supplementary Fig. 2a). The expression level of integrin αV/β8, GARP, and TGFβ was positively correlated with that of essentially all the macrophage phenotypic marker genes tested (CD86, CD163, CD204, CD206, CD209, CSF-1R) (Pearson *R* > 0.4, *P* value <0.01), but not or weakly correlated with lymphocyte markers tested (Pearson *R* < 0.4) (Supplementary Fig. 2b). The expression of integrin subunits αV/β8, GARP, and TGFβ−1 was also positively correlated with the infiltration of macrophages and in general was more strongly correlated with the infiltration of macrophages than that of T and B lymphocytes (Supplementary Fig. 2c). Taken together, these results supported a role of GARP/TGF-βRII on macrophages and integrin αV/β8 on PDA cells in mediating the macrophage–tumor interaction.

To further test the above hypothesis, we investigated the involvement of the TGF-β signaling pathway by searching for the source of TGF-β. We conducted TGFβ−1 ELISA assay to examine the secretion of TGFβ−1 by M0, M1-like, M2-like macrophages, and mouse KPC PDA cells. We found that M0, M1-like, M2-like macrophages, and PDA cells all secreted TGFβ−1 while PDA cells appear to be the major source (Supplementary Fig. 1g). Using flow cytometry analysis, we further demonstrated that the binding of biotin-conjugated TGFβ−1 to M1-like macrophages, but not to M0 and M2-like macrophages, was significantly increased after co-culturing with PDA cells compared to singly-cultured macrophages (Supplementary Fig. 1e). It should be noted that we cannot distinguish between TGF-β that bound to TGF-βR from that bound to GARP. Nevertheless, this result suggested that more TGF-β receptors on M1-like macrophages bound exogenous TGFβ−1 after the latent TGF-β was released from GARP upon co-culturing with PDA cells.

### M1-like, but not M2-like macrophages can be selectively reprogrammed by tumor-induced methylation

In light of the above findings, we next sought to understand the difference in phenotypical changes between M1-like and M2-like macrophages upon interacting with PDA cells. The results showed that macrophages isolated from M1-like macrophages co-cultured with mouse KPC PDA cells exhibited significantly decreased expression of M1 marker, while *CD16* remained unchanged. All above M2 marker genes except *Ym1/2* and *Ccl-2* had significantly increased expression, while *Ccl-2* also had an upward tendency (Fig. 3a, b). By contrast, both M1 and M2 markers in M2-like macrophages had an increased expression after co-culturing with PDA cells (Supplementary Fig. 3a). Therefore, these results suggest PDA cells selectively reprogram M1-like macrophages and, more importantly, PDA cells induce a phenotypical change of M1-like macrophages to a M2-like phenotype. On another hand, M2-like macrophages co-cultured with PDA cells remain to have a M2-like phenotype with an increased expression of M2 markers, likely secondary to the effect of tumor-derived TGFβ-1 as previously suggested.^27^ Nevertheless, the mechanism for the upregulation of some of the M1 markers in M2-like macrophages remains to be elucidated.

We next examined whether the tumor-induced metabolic reprogramming through gene methylation occurs in both M1-like and M2-like macrophages or selectively in M1-like macrophages. Methylation of *Nqo-1* and *Aldh1a3* was induced in M0 (unpolarized BMDMs) and M1-like macrophages, but not in M2-like macrophages, after co-cultured with mouse PDA cells (Fig. 3c). All the glucose metabolism genes were significantly downregulated in M1-like macrophages after co-culturing with PDA cells, except *Uqcrq* and *Gstp-1* (Fig. 3d). *Uqcrq* had a downregulated trend without a significant change. Such a metabolism gene expression change was not observed in M2-like macrophages. Many of the metabolism genes tested including *Nqo-1* and *Aldh1a3* were significantly upregulated in M2-like macrophages after co-culturing with PDA cells (Supplementary Fig. 3b).

Similarly, human PBMC derived macrophages were polarized to M1-like macrophages by stimulation with IFN-γ and LPS; and the majority of M1 markers were induced, including *CD86*, *TLR-2*, *TLR-4*, and *IL-6* (Fig. 3e). M2-like macrophages were induced by IL-4, IL-10, and TGF-β; and the majority of M2 markers were induced, including *CD206*, *IL-10*, and *CD209* (Fig. 3e). In M1-like macrophages, expression of all the M2 marker genes except *CD209* were increased following co-culturing with human PDA tumor cells, including those markers that were not induced by the above M2 cytokines (Fig. 3f). Different from mouse M1-like macrophages, M1 markers in human M1-like macrophages were not significantly suppressed following co-culturing with PDA cells. The difference in human and mouse macrophages may be due to the variance of human donors’ genetics, age, and environmental exposure. Nevertheless, the overall trend still suggests that PDA cells reprogram M1-like macrophages into phenotypically M2-like macrophages. Similarly, neither M1 markers nor M2 markers in human M2-like macrophages were as significantly affected by the co-culture with PDA cells as those in M1-like macrophages (Supplementary Fig. 3c). Moreover, methylation of *NQO-1* and *ALDH1a3* was induced in human M1-like macrophages, but not in human M2-like macrophages, after co-culturing with human PDA cells (Fig. 3g). Furthermore, all the metabolic genes tested were significantly downregulated in human M1-like macrophages, but not in human M2-like macrophages after co-culturing with human PDA tumor cells (Fig. 3h and Supplementary Fig. 3d). It should be noted that M2 polarization was not as complete as M1 polarization (Fig. 3e); therefore, *LDHC* and *COX7B2* were downregulated in M2-like macrophages co-cultured with PDA cells likely due to the presence of unpolarized M0 macrophages (Supplementary Fig. 3d). Nevertheless, taken together, our results suggest that PDA cells have the capacity of reprogramming macrophages by regulating the expression of metabolism genes through regulating their DNA methylation.

### GARP mediates the induction of DNA methylation in the *Nqo-1* gene and M2-like phenotypical changes in M1-like macrophages after co-culturing with PDA cells

**Fig. 4:**
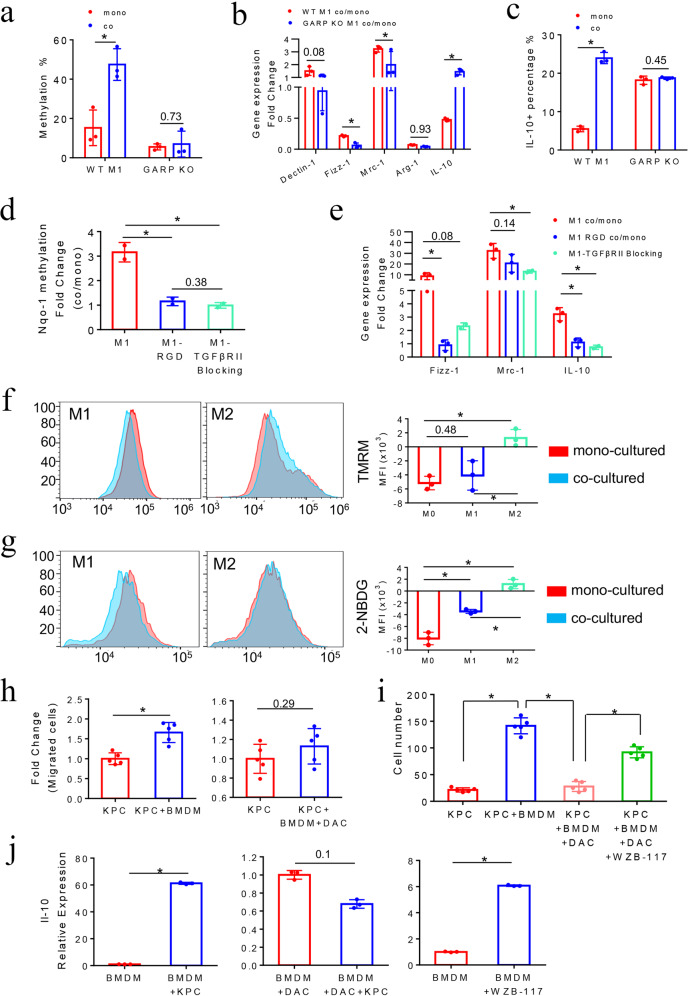
GARP mediates *Nqo-1* methylation and M2-like phenotypical changes in M1-like macrophages after co-culturing with PDA cells. **a** *Nqo-1* methylation in mouse WT M1-like macrophages compared to GARP KO M1-like macrophages. *P < 0.05 (paired *t* test). **b** RT-PCR of M2 marker genes in WT vs. GARP KO M1-like macrophages after co-cultured with KPC cells. Fold changes of these marker genes in co-cultured vs. monocultured M1-like macrophages were shown. Fold change >1: upregulation; fold change <1: downregulation. All results were first normalized by respective *β-actin* and then respective monocultured BMDMs. *P < 0.05 (Mann–Whitney *U* test). **c** Expression of the M2 cytokine IL-10 in WT vs. GARP KO M1-like macrophages after co-culturing with KPC cells, measured by flow cytometry analysis of percentages of IL-10-positive cells with intracellular staining of IL-10. *P < 0.05 (Mann–Whitney *U* test). **d** Fold changes of MSP results of the *Nqo-1* gene in co**-**cultured vs. monocultured M1*-*like macrophages treated with RGD or TGF-βRII blocking antibody. *P < 0.05 (ANOVA). **e** Fold changes of RT-PCR results of M2 marker genes in co-cultur**e**d vs. monocultured M1-like macrophages treated with RGD or TGF-βRII blocking antibody. Data were first normalized by respective β-actin and then respective monocultured M0 macrophages. *P < 0.05 (ANOVA). **f** Mitochondrial membrane potentials in mouse M0, M1-like and M2-like macrophages after co-culturing with KPC cells by measuring mean fluorescence intensity of TMRM signals on the PE channel of flow cytometry, comparing mono- vs. co-cultured macrophages. *P < 0.05 (ANOVA). **g** Glucose uptake activities in M0, M1-like, and M2-like macrophages by measuring mean fluorescence intensity of 2-NBDG signals, comparing mono- vs. co-cultured macrophages. *P < 0.05 (ANOVA). **h** KPC cells were co-cultured with mouse BMDMs or DAC pretreated BMDMs in upper chamber of a transwell system with 8-μm pore membrane that allows them migrating to the lower chamber. Migrated KPC cells were examined by immunofluorescent staining with FITC-conjugated anti-Pan-CK antibody and counted. Fold changes of migrated KPC cell number in co-cultured vs. monocultured group (normalized as 1) were shown. *P < 0.05 (Mann–Whitney *U* test). **i** KPC cells were co-cultured with BMDMs pretreated with DAC, glucose uptake inhibitor WZB-117, or DAC + WZB-117, respectively, in the transwell system. Numbers of migrated KPC cells were counted as described in (**h**) and shown. *P < 0.05 (ANOVA). **j** *Il-10* expression per RT-PCR in untreated, DAC, or WZB-117 pretreated BMDMs before (normalized as 1) and after co-culturing with KPC cells. *P < 0.05 (Mann–Whitney *U* test). Data are means ± SEM from technical duplicates and representative of two experiments

To further assess the tumor-induced DNA methylation in M1-like macrophages as well as the role of GARP in mediating macrophage–tumor interaction to induce DNA methylation changes, we employed BMDMs from *Lrrc32* (which encoded GARP) knockout mice. Supporting the role of GARP in mediating the macrophage–tumor interaction, M1-like macrophages derived from GARP knockout mice (GARP KO M1) did not demonstrate the induction of methylation in the *Nqo-1* gene by mouse KPC PDA cells comparing to wild-type M1-like macrophages (WT M1) (Fig. 4a). In addition, GARP KO M1-like macrophages had a deceased tendency of being reprogrammed to M2-like macrophages phenotypically by PDA cells compared to WT M1-like macrophages (Fig. 4b). The M2 marker gene *Dectin-1* has an increased expression in WT M1-like macrophages following co-culturing with PDA cells; however, it was inhibited in GARP KO M1-like macrophages following co-culturing with PDA cells. *Mrc-1* has an increased expression in WT M1-like macrophages following co-culturing with PDA cells, but was significantly less upregulated in GARP KO M1-like macrophages following co-culturing with PDA cells. Although *Fizz-1* and *Arg-1* were both downregulated in WT M1-like macrophages following co-culturing with PDA cells, they were more downregulated in GARP KO M1-like macrophages following co-culturing with PDA cells. The main exception was the *Il-10* gene. Previously, it was found that blocking glycolysis activities in macrophages did not have a significant effect on the *Il-10* mRNA expression.^17^ Therefore, we analyzed the IL-10 expression at the protein level by flow cytometry. After co-culturing with PDA cells, GARP KO M1-like macrophages failed to increase the expression of IL-10 (Fig. 4c), suggesting the tumor-induced IL-10 overexpression in WT M1-like macrophages is also attributed to a post-transcriptional mechanism. The upregulation at the mRNA level of *Il-10* observed in GARP KO M1-like macrophages following co-culturing with PDA cells may be a negative feedback as a result of suppressing IL-10 at the protein level. We also observed a higher baseline percentage of IL-10-expressing macrophages among GARP KO M1-like macrophages comparing to WT M1-like macrophages, suggesting that GARP may play a role in regulating the IL-10 protein expression in macrophages.

*Nqo-1* methylation was not induced in M1-like macrophages after co-culturing with KPC PDA cells in presence of the integrin-blocking arginine–glycine–aspartic acid (RGD) peptide or the antibodies blocking TGF-βRII on M1-like macrophages, respectively (Fig. 4d). The RGD motif is the GARP-binding motif on integrin αV/β8; and RGD peptides have been commonly used to block the interaction between integrin αV/β8 and GARP.^28,29^ This result supported the role of integrin in mediating the interaction between PDA cells and M1-like macrophages and the role of TGF-βR in inducing the intracellular signaling that ultimately leads to the methylation of the *Nqo-1* gene. Consistently, M2 marker genes such as *Fizz-1*, *Mrc-1*, and *Il-10* were significantly less upregulated in M1-like macrophages following co-culturing with PDA cells in presence of RGD peptides and TGF-βRII-blocking antibodies compared to M1-like macrophage following co-culturing with PDA cells in absence of RGD peptides and TGF-βRII-blocking antibodies (Fig. 4e).

### PDA cells induce DNA methylation and downregulation of metabolism genes more selectively in M1-like macrophages than in M2-like macrophages

To further demonstrate the metabolic reprogramming of TAMs is mediated by DNA methylation, we examined DNA methylation in metabolism genes beyond *ALDH1a3* and *NQO-1*. We did whole-genome methylation array analysis on human PBMC derived M0, M1-like, and M2-like macrophages, respectively, comparing to those following co-culturing with human PDA cells. In addition, we performed whole-transcriptomic analysis by RNA-seq on the same samples. DNA methylation array showed that 3352 genes, 3542 genes, and 2229 genes were methylated in M0, M1-like, and M2-like macrophages, respectively, after co-culturing with human PDA cells. This result suggests that tumor-induced methylation occurs globally in macrophages although more genes are methylated in M0 and M1-like macrophages than M2-like macrophages. Nevertheless, we found those genes in the glucose metabolism pathway and OXPHOS pathway as above described, but not other metabolism pathways, were more selectively methylated and downregulated in M1-like macrophages than M2-like macrophages after co-culturing with PDA cells. It should be noted that these genes are clustered on chromosomes as previously shown in CAFs,^5^ suggesting that specific chromatin remodeling mechanisms, likely downstreaming of GARP/TGFβ, are involved for the tumor-induced methylation of metabolism genes in M1-like TAMs and CAFs. As summarized in Table 1, 16 out of 20 *ALDH1a3*-related genes were methylated and the remaining 4 non-methylated genes were either downregulated or not detectable by RNA-seq in M1-like macrophages after co-culturing with PDA cells, whereas only 4 out of these 20 *ALDH1a3*-related genes were methylated in M2-like macrophages. Among 16 *ALDH1a3*-related genes methylated in M1-like macrophages after co-culturing with PDA cells, 7 were also downregulated at the mRNA level by >twofold. Moreover, 14 out of 15 *NQO-1*-related genes were methylated in M1-like macrophages after co-culturing with PDA cells whereas only 3 out of these 14 *NQO-1*-related genes were methylated in M2-like macrophages. Among 14 *NQO-1*-related genes methylated in M1-like macrophages after co-culturing with PDA cells, 9 were downregulated by >twofold. Minor inconsistency between RT-PCR and RNA-seq results (Supplementary Table 2) is likely due to the variation of RNA-seq or variation between macrophages from different donors. *ALDH1a3* was upregulated after co-culturing with PDA cells in M0 and M1-like macrophages (Supplementary Table 2), suggesting that *ALDH1a3* is regulated by a more complex mechanism. Nevertheless, methylation of *ALDH1a3* and *NQO-1* was induced in both human M0 and M1-like macrophages (Supplementary Fig. 4b and Fig. 3g).

**Table 1:** Summary of the analysis of the 35 metabolism genes in human M0, M1-like, and M2-like macrophages upon interacting with PDA tumor cells

ALDH1a3-related genes (genes in the glucose metabolism pathway)			ALDH3A1	FAHD1	GSTP1	ACADM	HK3
	M0 vs. M0.CO	DNA methylation Array	- ^A^	–	M	–	M
		RNA sequence	/^B^	↓	↓	↓	↓
	M1 vs. M1.CO	DNA methylation Array	M^C^	–	M	M	M
		RNA sequence	↓^D^	↓	↓	↑	↓
	M2 vs. M2.CO	DNA methylation Array	–	–	–	–	M
		RNA sequence	/	↑^E^	↓	↓	↓
			HK1	PFKP	ALDOA	GAPDHS	PGK1
	M0 vs. M0.CO	DNA methylation Array	M	–	M	–	–
		RNA sequence	↓	↑	↓	/	↓
	M1 vs. M1.CO	DNA methylation Array	M	M	M	M	–
		RNA sequence	↑	↑	↑	/	↓
	M2 vs. M2.CO	DNA methylation Array	M	–	M	–	–
		RNA sequence	↑	↓	↑	/	↑
			PGAM1	LDHC	LDHB		
	M0 vs. M0.CO	DNA methylation Array	–	–	M		
		RNA Sequence	↓	/	↓		
	M1 vs. M1.CO	DNA methylation Array	M	M	M		
		RNA sequence	↑	↓	↓		
	M2 vs. M2.CO	DNA methylation Array	–	–	M		
		RNA sequence	↑	/	↓		
ALDH1a3-related genes (genes in other metabolism pathways)			TPO	GSTM4	GSTK1	GSTA4	UGT2A3
	M0 vs. M0.CO	DNA methylation Array	–	–	–	M	–
		RNA sequence	/	↓	↓	/	/
	M1 vs. M1.CO	DNA methylation Array	M	M	M	M	–
		RNA sequence	/	↓	↓	/	/
	M2 vs. M2.CO	DNA methylation Array	–	–	–	–	–
		RNA sequence	/	↓	–	/	↑
			CYP3A43	ABAT			
	M0 vs. M0.CO	DNA methylation Array	–	–			
		RNA sequence	/	/			
	M1 vs. M1.CO	DNA methylation Array	–	M			
		RNA sequence	/	↑			
	M2 vs. M2.CO	DNA methylation Array	–	–			
		RNA sequence	/	↓			
NQO-1 related genes (genes in the OXPHOS pathways)			NDUFS6	NDUFA10	NDUFA12	NDUFB9	UQCRQ
	M0 vs. M0.CO	DNA methylation Array	–	–	–	–	–
		RNA sequence	↓	↓	↓	↓	↓
	M1 vs. M1.CO	DNA methylation Array	M	M	M	M	–
		RNA sequence	↓	↓	↓	↓	↓
	M2 vs. M2.CO	DNA methylation Array	–	–	–	–	–
		RNA sequence	↑	↑	↓	↑	↑
			COX7A1	COX7B2	ATP5D	ATP5G2	ATP6V1A
	M0 vs. M0.CO	DNA methylation Array	–	–	M	M	–
		RNA sequence	↑	/	↑	↓	↓
	M1 vs. M1.CO	DNA methylation Array	M	M	M	M	M
		RNA sequence	↓	/	↑	↑	↓
	M2 vs. M2.CO	DNA methylation Array	–	–	M	M	–
		RNA sequence	↓	/	↑	↓	↓
			ATP6V1E1	ATP6V0D1	ATP6V0D2	ATP6V0E1	ATP4A
	M0 vs. M0.CO	DNA methylation Array	–	M	–	–	–
		RNA sequence	↓	↓	↓	↓	/
	M1 vs. M1.CO	DNA methylation Array	M	M	M	M	M
		RNA sequence	↑	↑	↓	↓	/
	M2 vs. M2.CO	DNA methylation Array	–	–	–	–	M
		RNA sequence	↓	↓	↓	↓	/

^A^-: no significant change, ^B^/: not performed, ^**C**^M: methylation; ↑; ^**D**^ ↓ : downregulated; ^**E**^ ↑ **:** upregulated

Thus, the above results support a reprogramming of glucose metabolism and OXPHOS genes in TAMs through tumor-induced DNA methylation. We further studied the glucose metabolic states of macrophages after co-culturing with PDA cells by using TMRM to measure mitochondrial membrane potential and 2-NBDG, a glucose analog to measure glucose intake. Higher TMRM and 2-NBDG signals suggested higher OXPHOS activities and glycolysis activities, respectively. M1-like and M0 macrophages showed decreased TMRM and 2-NBDG signals after co-culturing with mouse PDA cells compared to their monocultured counterparts, respectively, consistent with the above-observed downregulation of glucose metabolism and OXPHOS genes (Fig. 4f, g). We also observed slightly higher TMRM and 2-NBDG signals in M2-like macrophages after co-culturing with PDA cells compared to the monocultured M2-like macrophages. Some of the glycolysis-related genes tested were upregulated in M2-like macrophages following co-culturing with PDA cells (Supplementary Fig. 3b, d), possibly due to a slightly higher 2-NBDG level in M2-like macrophages than in M1-like macrophages. Taken together, our results suggest that PDA cells mainly induce the metabolic reprogramming of M1-like and M0 macrophages. Such a metabolic reprogramming process correlates with tumor-induced DNA methylation on metabolic genes in M1-like and M0 macrophages.

### Macrophages can promote PDA cell migration in the co-culture system in a DNA methylation-dependent manner

We then wondered whether the phenotypical and metabolic reprogramming would lead to the functional reprogramming of macrophages. To this end, we examined whether phenotypical reprogramming of macrophages can convert the function of macrophages from anti-tumoral to protumoral. As TAMs play a role in tumor migration and metastasis,^12^ we examined whether M0 macrophages from BMDMs can promote mouse KPC PDA cell migration in vitro in a transwell migration assay (Supplementary Fig. 1a). To quantify migrated PDA cells and macrophages, we used Pan-CK staining to mark PDA cells and used F4/80 staining to mark macrophages. Most of PDA cells co-migrated with macrophages (Supplementary Fig. 1b). Co-culturing with BMDMs led to a significant increase of migrated PDA cells compared to monocultured PDA cells (Fig. 4h). We next examined whether macrophage-mediated pro-migration of PDA cells can be reversed by blocking tumor-induced methylation in macrophages. DAC and glucose uptake inhibitor (WZB-117) treatment has no significant effect on the cell viability of BMDMs (Supplementary Fig. 4f). The pro-migration effect of macrophages on PDA cells was abolished by pretreating BMDMs with DAC (Fig. 4h). Although cell viability of BMDMs was decreased after the sequential treatment of DAC and WZB-117 (Supplementary Fig. 4f), pretreatment of BMDMs with WZB-117 reversed the effect of DAC on BMDMs (Fig. 4i). These results suggested that macrophages promote PDA cell migration likely by reprogramming the DNA methylation of glucose metabolism genes in macrophages.

### Tumor-educated macrophages promote PDA cells migration through the *IL-10*/*IL-10R* pathway as a result of glucose metabolism reprogramming

To understand how tumor-educated macrophages promote tumor cell migration through glucose metabolism reprogramming, we sought to identify glucose-response genes in macrophages. Previously, a whole-transcriptomic analysis identified 100 glucose-response genes either increased or decreased after attenuating LPS-induced macrophage activation.^30^ Among them, we selected 23 genes known to be functionally relevant to PDA, including either anti-tumoral or protumoral genes (Supplementary Fig. 5a). Conceiving a paracrine mechanism, ten genes whose products are either expressed on the cell surface or secreted were selected for further study, including *Mt-1, Il-1b, Ccr-5, MerTK, Adam-8, Ccl-7, Il-10, Batf-2, Txnip*, and *Emp-1* (Supplementary Fig. 5a–d). We found that *Il-10* mRNA expression in mouse BMDMs was increased after the WZB-117 treatment or co-culturing with PDA cells; however, this increase was reversed by DAC pretreatment (Supplementary Fig. 5d and Fig. 4j).

To examine whether TAMs promote tumor migration through the *Il-10*/*Il-10R* pathway, we examined tumor cell migration using the transwell assay. As it is not feasible to maintain the M1-like macrophage phenotype during the long course of pretreatment with DAC, WZB-117 or lentiviral vectors, we used unpolarized mouse unpolarized BMDMs (M0 macrophages) for the transwell assay since it has a similar tumor-induced methylation as M1-like macrophages. We used lentivirus-carried shRNA to *Il-10* expression in macrophages and *Il-10Ra* in mouse KPC PDA cells (Supplementary Fig. 5e). Knockdown of *Il-10* and *Il-10Ra* has no significant effect on the cell viability of BMDMs or PDA cells, respectively (Supplementary Fig. 5f). PDA cell migration was significantly inhibited by *Il-10* knockdown from macrophages or *Il-10Ra* knockdown from PDA cells (Supplementary Fig. 5g). These results suggested that PDA cells stimulate the production of a glucose-response gene *Il-10* in macrophages in a DNA methylation-dependent manner to promote their migration through the IL-10 receptor on them. Other glucose-response genes may also be involved and warrant further investigation.

### Tumor-educated macrophages promote tumor metastasis in “orthotopic” mouse model of PDA in a DNA methylation-dependent manner

**Fig. 5:**
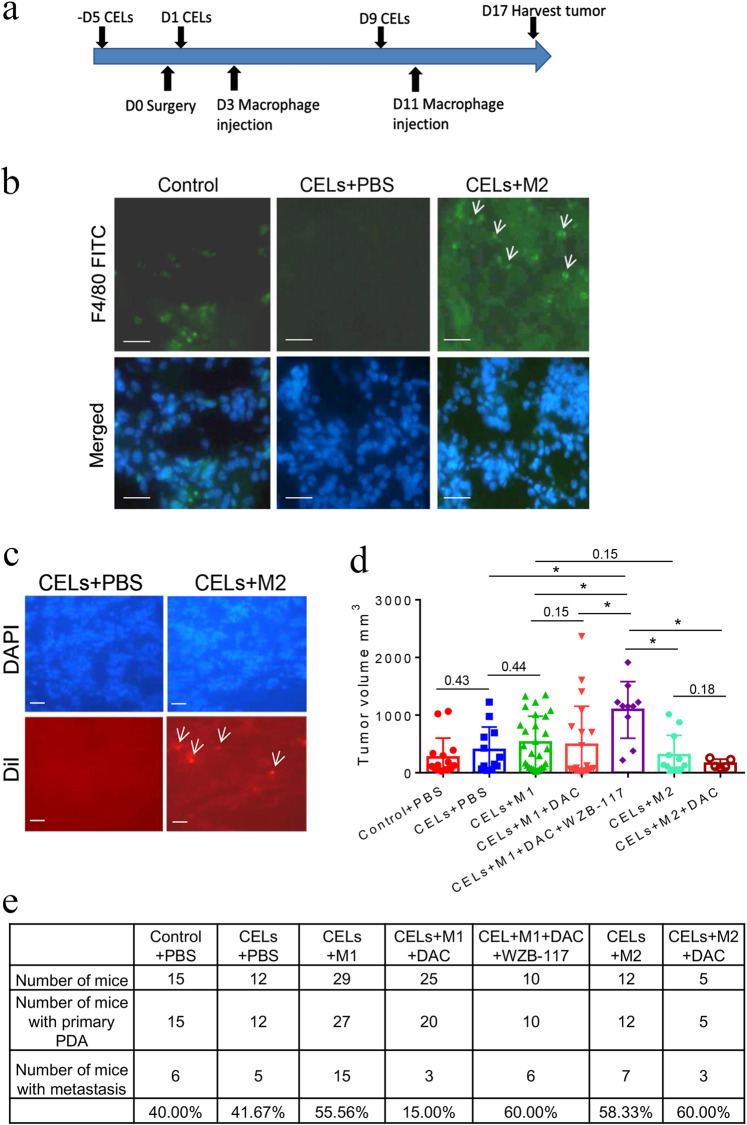
Tumor-educated macrophages promote metastasis in the “orthotopic” mouse model of PDA in a DNA methylation-dependent manner. **a** Scheme of the experiment. The exogenous macrophages were isolated from eight C57BL/6 mice. **b** Pancreas tissues were stained with FITC-conjugated anti-F4/80 antibody to evaluate macrophage depletion and exogenous macrophage infusion. Representative treatment groups are shown. Arrow indicates F4/80-positive macrophages. Scale bar: 50 μm. **c** Examination of exogenous macrophages pre-labeled by Vybrant Dil. Arrow indicates Vybrant Dil-positive macrophages in pancreas tissues. Scale bar: 50 μm. **d** Average sizes of pancreatic tumors at the end of the experiments in mice of each treatment group as indicated. Data are means ± SEM from triplicates, *P < 0.05 (ANOVA). **e** Percentag**e**s of mice that had metastasis in each treatment group in (**d**). Primary tumors and metastases were examined both grossly and microscopically. Note that some primary tumors were not visualized grossly likely due to technical variations of tumor implantation. DAC + M1 vs. other CELs groups (*P* = 0.002), DAC + M1 vs. DAC + WZB-117 + M1 (*P* = 0.03). CELs+M1 vs. CELs+ M2 (*P* = 0.65). All used Fisher’s exact tests

We next examined whether tumor-educated macrophages promote PDA metastasis in vivo as a result of glucose metabolism reprogramming in a DNA methylation-dependent manner. Here, we used more physiologically relevant, immunocompetent, a syngeneic mouse model with “orthotopic” PDA implantation and depletion of endogenous macrophages with Clodronate-containing liposomes (CELs) followed by exogenous macrophage infusion (Fig. 5a). Pancreas tissues were stained with F4/80 to evaluate the efficiency of macrophage depletion (Fig. 5b). Homing efficiency of exogenous macrophages labeled by Vybrant Dil to the pancreas was similar among all groups (Fig. 5c and Supplementary Fig. 6a, b). All mice were sacrificed 17 days following tumor implantation. The average size of pancreatic tumors in the CELs+M1 + DAC + WZB-117 group infused with exogenous M1-like macrophages pretreated with DAC and WZB-117 was significantly larger than the other six groups; however, there was no significant difference in the size of the pancreatic tumors among these six groups, suggesting that the effect of macrophage reprogramming is not primarily in the primary tumor growth (Fig. 5d). As previously observed with this model,^31^ mice primarily developed liver and peritoneal metastasis (Supplementary Fig. 7a, b). The CELs+M1 group infused with exogenous M1-like macrophages following CEL treatment developed metastases as frequently as the CELs+M2 group, suggesting that the exogenous M1-like macrophages were reprogrammed by tumor cells and thus have lost their antitumor functions. However, significantly fewer mice in the CELs+M1 + DAC group infused with DAC pretreated M1-like macrophage following CEL treatment developed metastases than any other group (Fig. 5e). Mice with macrophage depletion (CELs + PBS) exhibited a metastasis rate similar to that of the control+PBS group, suggesting that macrophages may have a tumor-restricting role; thus, depletion of recruited macrophages would also permit tumor metastasis, an observation seen similarly as a result of CAF depletion.^3,4^ This experiment was repeated multiple times. More mice were included in the CELs+M1 group and CELs+M1 + DAC group because they were two main comparison groups. Accumulatively, 3 out of 25 mice in the CELs+M1 + DAC group developed metastasis. By contrast, pretreatment of M1-like macrophages with the glucose uptake inhibitor, WZB-117, in addition to DAC, abolished the effect of pretreatment of DAC. In this CELs+M1 + DAC + WZB-117 group, six out of ten mice developed metastases (Fig. 5e). This result further suggested that M1-like macrophages would originally have the antitumor capacity, however, have acquired protumoral and pro-metastasis function in a DNA methylation-dependent manner after being reprogrammed by tumor cells. Direct glucose metabolism inhibition triumphs the effect of DNA demethylation, suggesting that inhibiting glucose metabolism directly will mimic the effect of the metabolism gene methylation that is induced by tumor cells.

### TAMs from mouse PDA have downregulated gene expression of the metabolism genes

**Fig. 6:**
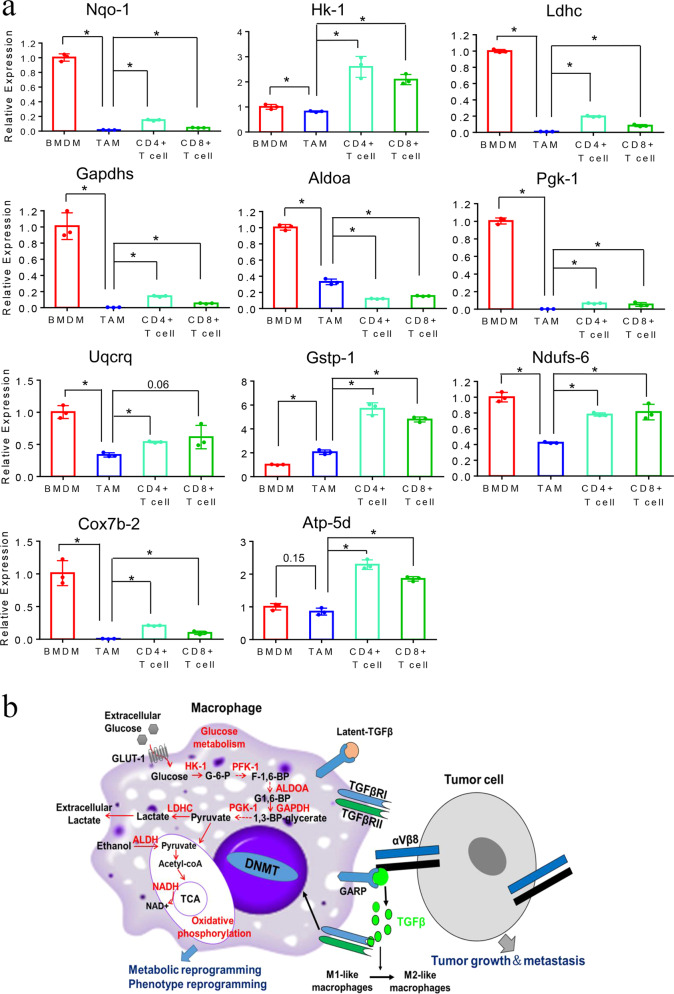
Downregulation of genes in the metabolic pathway in TAMs from murine PDA. **a** mRNA expression of metabolism genes as indicated were measured by RT-PCR in TAMs, CD4^+^, and CD8^+^ T cells from primary pancreatic tumors and BMDMs of the same KPC mice. Tumors were identified by ultrasound before sacrifice. *β-actin* used for normalization. Data are means ± SEM from triplicates and representative of two experiments. *P < 0.05 (ANOVA). **b** The schematic model of the GARP/integrin-mediated interaction between tumor cells and macrophages in the TME of PDAC and the mechanisms of metabolic, phenotypical, and functional reprogramming of macrophages from M1-like to M2-like macrophages in a DNA methylation-dependent manner

We next sought to explore the metabolic reprogramming of TAMs in vivo in spontaneously developed PDA in KPC mice. We examined the metabolic gene expression in BMDMs, TAMs, CD4^+^, and CD8^+^ T cells, respectively. We anticipated that the expression of glucose metabolism and OXPHOS genes would be similar to the above described tumor-educated M1-like macrophages. TAMs, intratumoral CD4^+^ T cells, CD8^+^ T cells, and BMDMs were isolated from the same mice. We chose 11 genes in the glucose metabolism pathway and OXPHOS pathway showing downregulation in mouse BMDMs after co-culturing with PDA cells. We found that all these 11 genes except *Gstp-1* and *Atp-5d* were significantly downregulated in TAMs, but not in intratumoral CD4^+^ and CD8^+^ T cells, compared with BMDMs from the same mouse (Fig. 6a). *Atp-5d* was also downregulated in TAMs compared with BMDMs in a non-statistically significant trend. The expression of *Gstp-1* in TAMs was higher than that in BMDMs and was significantly lower than that in intratumoral CD4^+^ T cells or CD8^+^ T cells. Taken together, these results further supported the hypothesis that glucose metabolism genes in macrophages are reprogrammed in vivo in PDAs.

## Discussion

Our study for the first time demonstrated that PDA cells can reprogram the function of macrophages within the TME by inducing the methylation of the glucose metabolism and OXPHOS genes. This reprogramming process occurs selectively in M1-like macrophages, but not in M2-like macrophages. Genes in glucose metabolism and OXPHOS pathways were suppressed in tumor-educated M1-like macrophages, subsequently leading to the metabolic reprogramming and the functional reprogramming of M1-like macrophages. Macrophages are known to have the capability of rapidly increasing cytokine and chemokine synthesis and release in response to the reprogramming process.^8^ Consistently, our study showed that *IL-10* expression is increased significantly in macrophages through this reprogramming mechanism. *IL-10* is conceived as an immunosuppressive cytokine that favors tumor escape from immune surveillance.^32^ *IL-10* has also been shown to have tumor-promoting functions.^32^ In addition, *IL-10R* is found to be upregulated in metastatic tumors and crucial for cancer cell migration.^32^ Our study thus revealed a novel mechanism of reprogramming M1-like macrophages to express a M2 cytokine, IL-10 that interacts with its receptor on the tumor cell surface and subsequently activates the IL-10/IL-10R-downstream signaling in tumor cells to promote metastasis and also revealed a novel mechanism of PDA metastasis through reprogramming macrophages from M1-like to M2-like macrophages by tumor cells to permit their metastasis (Fig. 6b). By contrast, M2-like macrophages, which are anticipated to have already had pro-cancerous activities, are not reprogrammed by tumor cells according to our study. Therefore, PDA tumors appear to have the capability of selectively reprogramming the antitumor component of TME to promote their development and metastasis. These findings also explain why TAMs exhibit predominantly M2 phenotypes while new, unpolarized BMDMs are anticipated to be constantly recruited to neoplastic tissues.

It should be noted that the methylation array analysis or the RNA-sequencing analysis may not capture all the methylated sites or downregulated genes, respectively. We cannot exclude the possibility of methylation and/or downregulation of additional genes in M1-like macrophages after co-culturing with PDA tumor cells if their methylation or RNA expression could be captured by methylation array analysis or RNA-sequencing analysis, respectively. On another hand, the *ALDH1a3* gene is induced, but its expression is not reduced, in M0 and M1-like macrophages after co-culturing with human PDA tumor cells. *ALDH1A3* was shown to activate *PPARγ* and increase the expression of *HK2*, which subsequently promotes glycolysis.^33^ Thus, *ALDH1a3* is not essential for the glycolysis pathway. This may explain why methylation of the *ALDH1a3* gene is induced, but its expression is not reduced, in M0 and M1-like macrophages after co-culturing with human PDA tumor cells. It is possible that *ALDH1a3* has a more complicated regulatory mechanism during macrophage polarization. This study is also limited by the lack of in vivo analysis of glucose metabolic enzymes and polarization markers in macrophages at the PDA tissue level. It is difficult to distinguish the expression of glucose metabolic enzymes and polarization markers in macrophages from that in other stromal cells. In the future, multiplex immunohistochemistry will be developed to evaluate glucose metabolic enzymes and polarization markers specifically in macrophages at the PDA tissue level.

The mechanism for tumor cells to selectively reprogram M1-like macrophages warrant further exploration in future studies, which will present a high potential for identifying critical new targets for anticancer therapies. Our study suggested direct cell–cell contact mediates the tumor-induced DNA methylation in M1-like macrophages, implicating that cell-surface receptor may determine the M1-like macrophage selectivity. We further identified GARP as the potential mediator of the direct contact between macrophages and PDA tumor cells. Integrin αV/β8, the binding partner of GARP, is expressed on PDA tumor cells, suggesting that the mechanism for the release of latent TGF-β from GARP upon binding of integrin αV/β8 to GARP does exist (Fig. 6b). A previous report suggested that soluble GARP is involved in the polarization of M2 macrophages, which is consistent with the findings in this study showing the cell-surface-bound GARP in mediating the reprogramming of macrophages to acquire the M2 phenotypes in the neoplasms.^34^ Nevertheless, the reprogramming of macrophages by PDA tumor cells via a cell-to-cell direct contact would unlikely involve soluble GARP. It should be noted that M0 macrophages are not present in the tumor microenvironment. In our observation, M0 macrophages behave similar to M1-like macrophages upon interacting with tumor cells. However, we did not observe an increase of GARP expression in M0 macrophages as significantly as M1-like macrophages. It is possible that a modest expression of GARP expression in M0 macrophages would be sufficient to induce the DNA-demethylating process. Another possibility is that M0 macrophages are transformed into M1-like macrophages upon co-culturing with tumor cells and subsequently express GARP to mediate the direct interaction with tumor cells. Further investigation of the mechanism that mediates the interaction between M0 macrophages and tumor cells will help elucidate the transformation that peripheral monocytic myeloid cells undergo when they are trafficking into the tumors.

Our study on metabolic reprogramming through a direct cell-to-cell contract mechanism complements a recently published study showing that macrophages can also be reprogrammed by PDA cells through a paracrine mechanism. However, the latter reprogramming mechanism regulates the nucleotide metabolism and thus affects the sensitivity of PDA to certain chemotherapy agents such as gemcitabine.^18^ There is no evidence suggesting that such a nucleotide metabolic dysregulation in macrophages would modulate the polarization of macrophages or the function of macrophages in promoting cancer growth and metastasis. Nevertheless, it would be interesting to investigate whether the nucleotide metabolic dysregulation would also influence macrophage polarization. In addition, our study showed that cancer-reprogrammed M1-like macrophages exhibit downregulation of the OXPHOS genes. Therefore, M2-like macrophages in the neoplasms, which, suggested by the findings in this study, are reprogrammed from M1-like macrophages, are still different from M2-like macrophages under benign conditions. The latter is characterized by an increased demand of OXPHOS. These levels of downregulation in the glycolysis and OXPHOS processes do not appear to affect the viability of macrophages and essential intracellular activities in macrophages,^14,15^ instead, may modulate the intracellular signaling and gene expression in macrophages such as the expression of metabolism-responsive genes such as IL-10 shown in this study. Further development of the multiplex immunohistochemistry technique to include the staining of the functional markers of M1-like and M2-like macrophages, as well as the glucose metabolism and OXPHOS enzymes, will help distinguish between M2-like macrophages that are polarized by tumor cells through the reprogramming of M1-like macrophages and M2-like macrophages that are residential in the tumor tissues. Nevertheless, it would be intriguing to investigate whether tissue-resident macrophages are also reprogrammed to acquire M2-like phenotypes by neoplasms through a similar mechanism.

The mechanism in M1-like macrophages that determines DNA methylation in specific clusters of genes remains to be explored. A chromatin remodeling mechanism may be involved to recruit DNMTs to specific loci on chromatin to allow methylation specificity. Further identification of the factors that recruit DNMTs to specific chromatin loci is warranted and may also facilitate the development of therapeutics that targets the macrophage’s reprogramming process. Such a potential therapeutic strategy may be superior over the currently used global DNA-demethylating agents such as decitabine and azacytidine by targeting the methylation of specific clusters of genes. Future studies will further aim at targeting GARP to reverse the reprogramming process in M1-like macrophages without affecting other functions of macrophages. Targeting the cell-surface receptors that mediate this interaction is anticipated to have antitumor therapeutic benefits. Therapeutic agents targeting GARP and integrin αV/β8 are being developed and thus warrant being tested as a TAM-modulating strategy.^20^

A limitation of this study is the use of the phenotypic markers for the dichotomy classification of M1 and M2 macrophages, which are extreme phenotypes induced in vitro. This limitation is due to currently the lack of a functional classification of heterogeneous macrophage populations in the tumors. However, our data, by demonstrating an epigenetic, metabolic, and functional shift between phenotypically M1-like and M2-like macrophages in vitro and in vivo, have further supported the notion that the dichotomy classification of M1 and M2 macrophages is inappropriate. Our study may thus provide a clue for functionally defining different statuses of TAM in future studies.

In conclusion, this study shows that PDA tumor cells reprogram M1-like macrophages, leading to metabolism disturbance, phenotypic switch, and functional change. This reprogramming mechanism in turn promotes pancreatic cancer cell migration and metastasis.

## Materials and methods

### Mouse models of PDA

The KPC (Kras^G12D/+^; TP53^R172H/+^; Pdx-1-Cre^+/+^) mouse model is a genetically engineered mouse model of PDA, which was previously established through a knock-in of pancreatic-specific, conditional alleles of the Kras^G12D^ and TP53^R172H^ mutations, and spontaneously develops pancreatic malignancies that resemble human PDA.^35^ We have backcrossed the KPC mice to the C57BL/6 mice background.^31^ The “orthotopic” mouse syngeneic model of PDA in the C57/BL6 mouse was established, as described previously.^36^ Syngeneic C57BL/6 mice at ~8-weeks old were used for the in vivo tumor metastasis assay. In brief, 2 × 10^6^ mouse PDA cells were subcutaneously injected into two flanks of C57BL/6 mice. After 2 weeks, subcutaneously formed tumors were harvested and cut into 2-mm^3^ pieces for tumor implantation into the pancreas of C57BL/6 mice. All animal experiments were performed by following the guidelines of the Animal Care and Use Committee of the Johns Hopkins University; and animals were maintained in accordance with the AALAC guidelines. The long (*L*) and short (*S*) axes of each tumor were measured with calipers after being harvested on day 17. Tumor volume (*V*) was calculated as *V* = (*L* × *S*^2^)/2. The bone marrow cells were isolated from the KPC mice together with matched tumor-associated macrophages (TAMs) or from the healthy C57BL/6 mice according to the literature.^37^

### Cell lines and human tissues

The KPC PDA tumor cell line was developed from a KPC mouse as described previously.^31,36^ Human pancreatic cancer cell line Panc10.05 cell line was established in 1998 in accordance with the Johns Hopkins Medical Institution Institutional Review Board (JHMI IRB)-approved protocols and authenticated by DNA and gene expression profiling as previously described.^38^ Both KPC and Panc10.05 tumor cell lines were cultured in 10% FBS RPMI 1640 media. De-identified archived human PDA tissues were obtained according to the JHMI IRB-approved protocol (IRB00138853).

### Mouse bone marrow-derived macrophages (BMDMs) isolation and polarization

After isolating from the femur of the mouse, BMDMs were plated at 1 × 10^6^ cells per 10-cm dish (Corning Lifesciences, Tewksbury, MA, USA) with 10 ml of macrophage complete media (10% FBS RPMI 1640), containing 50 ng/ml mouse recombinant macrophage colony-stimulating factor (M-CSF; Biolegend, San Diego, CA, USA) and cultured for 7 days, with the change of media and M-CSF on day 5. Mouse BMDMs were treated with 400 ng/ml LPS (Sigma, St. Louis, MO, USA), 50 ng/ml IFN-γ (Biolegend) for 24 h for M1 polarization, and 50 ng/ml IL-4 (Biolegend) for M2 polarization on day 7.

### Human monocyte-derived macrophages isolation and polarization

Human PBMCs were isolated from buffy coats from healthy donors according to standard procedures. Monocytes were purified from PBMCs using CD14^+^ magnetic microbeads (Miltenyi Biotec, GL, GER). Thereafter, the monocytes were cultured in macrophage complete medium with 100 ng/ml human recombinant M-CSF (Biolegend) for 6 days. On day 6, macrophages were treated with either 100 ng/ml human recombinant GM-CSF (Biolegend), 400 ng/ml LPS (Sigma) and 50 ng/ml IFN-γ (Biolegend) for M1 polarization or 100 ng/ml human recombinant M-CSF (Biolegend), 50 ng/ml IL-4 (Biolegend), 50 ng/ml IL-10 (Biolegend) and 50 ng/ml IL-13 (Biolegend) for M2 polarization for 24 h.

### Tumor-associated macrophages (TAMs) purification

PDA tumor growth in KPC mice was monitored every month using small-animal ultrasound (Vevo770, VisualSonics, Toronto, Ontario, Canada). The tumor was harvested when reaching 10 mm in size, minced, and dissociated in pre-warmed digest medium [5% FBS RPMI 1640 with collagenase (1500 U/ml), and hyaluronidase (1000 U/ml); Life Technology, Carlsbad, CA, USA] and incubated at 37 °C for 1 h. Tumor cells after digestion were filtered through a cell strainer, centrifuged, and washed with cold PBS.

TAMs were sorted by the Flow Cytometer. The cell suspension was stained with a mixture of PE-conjugated anti-mouse F4/80 antibody (Biolegend), APC-conjugated anti-mouse CD3 antibody (Biolegend), PE-Cy™7 rat anti-mouse CD8a (BD Biosciences, San Jose, CA, USA), FITC-conjugated anti-CD4 antibody (Biolegend), and PI (BD Biosciences) for 30 min. CD3-F4/80^+^ live cells were selected for analysis.

### Macrophages depletion and reconstitution in vivo

C57BL/6 female mice were treated with 100 μl Clodronate-containing Liposomes (CELs) (Liposoma B.V. Amsterdam, Netherlands) three times by intraperitoneal injection to deplete the resident macrophages at 5 days prior to the tumor implantation, also on day 1 and day 9 post-tumor implantation. Mouse BMDMs were isolated and polarized into M1 or M2 macrophages in vitro as described. Polarized macrophages were stained with Vybrant^®^ DiI Cell-Labeling Solution (Thermo Scientific, Hudson, NH, USA) before being transferred to mice through intravenous injection.

### Tumor and macrophages co-culture

In total, 5 × 10^5^ mouse KPC PDA tumor cells and 5 × 10^5^ mouse BMDMs were plated at a ratio of 1:1 and seeded into one well of the six-well plate with a macrophage complete medium containing 50 ng/ml mouse M-CSF (Biolegend) and co-cultured for 24 h. Panc10.05 tumor cells and human PBMC derived macrophages were plated at a ratio of 1:1 and seeded into one well of the six-well plate with 100 ng/ml human recombinant M-CSF (M0 and M2 macrophages) (Biolegend) or 100 ng/ml human recombinant GM-CSF (M1 macrophages) (Biolegend) and co-cultured for 48 h. Both tumor-educated macrophages and monocultured macrophages were purified by CD11b^+^ magnetic microbeads (Miltenyi Biotec) according to the manufacture’s protocol. For the contact and non-contact co-culture, 5 × 10^5^ mouse BMDMs and 5 × 10^5^ mouse KPC PDA tumor cells were co-cultured in the transwell system separated by an 8.0-µm or a 1.0-µm semitransparent membrane (Corning Lifesciences), respectively, for 1 or 3 days.

### Tumor-conditioned medium (TCM)

Mouse KPC PDA tumor cells were cultured in the macrophage complete medium for 48 h; and then TCM was collected and centrifuged at 3000× *g* for 5 min to remove the cells. The supernatant was filtered through a 0.22-μm polyethersulfone membrane (Corning Lifesciences). Ten times concentrated TCM was obtained by centrifuging TCM at 3500×*g* in Centricon^®^ Plus-70 Centrifugal Filter Units (EMD Millipore, Billerica, MA, USA) for 30 min.

### Cell proliferation assay

CCK-8 assay was used to assess cell proliferation. Cells were seeded at 5 × 10^3^ cells/well into a 96-well plate, followed by 24 h of culture at 37 °C. 1 μmol/L DAC, 2.5 μmol/L WZB-117, or shControl lentivirus and shIL-10 lentivirus, were added to the culture of BMDMs for an additional 24 h along with shControl lentivirus or shIL-10Ra lentivirus, respectively. After co-culture, the culture medium was removed, followed by the addition of CCK-8 reaction solution according to the manufacturer’s instructions. Relative cell viability was calculated in percentage based on the absorbance signal of the experimental group compared to that of the control group.

### Cell migration assay and immunofluorescence staining

Transwell migration assay was used to study the migratory capability of mouse KPC PDA tumor cells. Mouse BMDMs were treated with 1 μmol/L DAC (Sigma) or 2.5 μmol/L WZB-117 (Sigma) for 3 days prior to being seeded in the transwell. Fresh media without DAC or WZB-117 was used during the cell migration assay. In total, 2 × 10^5^ mouse KPC PDA tumor cells alone or with 2 × 10^5^ mouse BMDMs were cultured in the upper chamber with 8.0-µm pore polycarbonate membrane in the serum-free RPMI 1640 media, and macrophage complete medium was added to the bottom well. Unmigrated cells in the upper chamber were removed at 48 h. Cells migrated to lower chambers were fixed by 4% paraformaldehyde (Sigma), permeabilized by 0.4% Triton-X, blocked by 10% goat serum and incubated with PE-conjugated anti-pan-cytokeratin (pan-CK) antibody (clone C-11, EMD Millipore), FITC-conjugated anti-mouse F4/80 antibody (clone BM8, eBioscience, Hudson, NH, USA) according to the manufacturer’s instructions. Mouse KPC PDA tumor cells were transfected with either *Il-10R*a-targeted shRNA or control shRNA prior to being seeded in the transwell. Mouse BMDMs were transfected with either *Il-10*-targeted shRNA or control shRNA prior to being seeded in the transwell insert. The number of migrated KPC PDA tumor cells in each high-power field of microscope was counted after immunofluorescence staining. Ten fields of the microscope were chosen for each group.

### Cell surface and intracellular staining and flow cytometry analysis

Mouse M0, M1, M2 macrophages polarized from BMDMs were seeded in the wells of a 96-well plate at a volume of 100 μL per well at a density of 5 × 10^5^ cells/well. The cells in each well were stained with Live-Dead Aqua (Invitrogen) for 30 min on ice, washed, and then blocked with rat anti-mouse Fc antibody (CD16/CD32, clone 2.4G2, BD Biosciences) in FACS buffer for 10 min on ice. The FACS buffer consisted of HBSS (Sigma) buffer with 2% bovine calf serum (Sigma). FITC-conjugated anti-Glycoprotein A Repetitions Predominant (GARP) antibody (clone Plato-1, Enzo Life Sciences), APC-conjugated anti-transforming growth factor-beta type II receptor (TGF-βRII) antibody (R&D Systems), PE-conjugated anti-integrin ɑV antibody (clone RMV-7, Biolegend), and PE-conjugated anti-integrin β8 antibody (clone RMP1-30, eBioscience) were used for cell staining. For IL-10 intracellular staining, cells were permeabilized with the permeabilization kit (BD Biosciences) for 30 min on ice followed by intracellular staining with BV421-conjugated anti-mouse IL-10 antibody (Clone JES5-16E3, Biolegend) for 30 min on ice. For the TGF-β cell-surface staining, M0, M1, and M2 macrophages monocultured or co-cultured with KPC tumor cells were stained with biotinylated anti-TGF-β1 antibody (R&D Sytems) for 30 min on ice and then washed followed by staining with PE-conjugated streptavidin (BD Biosciences) for another 30 min on ice. All flow cytometry analyses were performed using the GytoFLEX flow cytometer (Beckman Coulter).

### TGF-β1 ELISA

Quantitative analysis of TGFβ−1 levels in the cell culture supernatant was performed by ELISA using commercially available kits (BMS623-3, Invitrogen Life Technologies). Mouse M0, M1, M2 macrophages were cultured in macrophage culture media without FBS. KPC tumor cells were cultured in KPC culture media without FBS. After incubation at 37 °C for 24 h, cell culture supernatant of M0, M1, M2 macrophages, and KPC tumor cells were collected and centrifuged, respectively. The supernatant was kept on ice. The concentration of TGFβ−1 in the cell culture supernatant was measured by the ELISA kit (BMS623-3, Invitrogen Life Technologies) according to the manufacturer’s instructions. The standard curve was made by using recombinant TGFβ−1 provided by the kit and used for normalization. The lowest detection limit of the assay was 31.25 pg/mL. The absorbance value was recorded at 450 nm.

### 5-Aza-2’-deoxycytidine (decitabine; DAC) treatment and WZB-117 treatment

In all, 5 × 10^6^ mice BMDMs were plated in 100-mm dish and treated with 1 μmol/L DAC (Sigma) or 2.5 μmol/L WZB-117(Sigma) for 3 days, fresh media with 1 μmol/L DAC or 2.5 μmol/L WZB-117 was changed every day.

### shRNA knockdown of *IL-10* and *IL-10Ra*

The vector expressing shRNA against the mouse *Il-10* or *Il-10R*a was obtained from Dharmacon (https://dharmacon.horizondiscovery.com/). The Lentivirus vector carrying sh*Il-10*, sh*Il-10R*a, or shControl was constructed and transfected into mouse BMDMs or KPC tumor cells as previously described.^36^

### Arginine–glycine–aspartic acid (RGD) peptides and anti-TGF-βRII antibody treatment

The KPC tumor cells were cultured in KPC culture media containing 230 μmol/L RGD peptides (BML-P700-0005, Enzo Life Sciences) for 48 h before co-culturing. Mouse BMDMs were cultured in macrophage culture media containing 2.5 μg/μl anti-TGF-βRII antibodies (AF-241-NA, R&D System)^39^ for 24 h prior to polarization. Fresh antibody was added along with polarization cytokines for another 24 h during polarization. Then M1 macrophages were co-cultured with KPC PDA tumor cells or in monoculture for 24 h. In all, 230 μmol/L RGD peptides or 2.5 μg/μl anti-TGF-βRII antibodies were added to the culture media during co-culture, respectively. Paired M1 macrophages in monoculture were also cultured in media containing 230 μmol/L RGD peptides or 2.5 μg/μl anti-TGF-βRII antibody as control.

### IHC staining

The anti-mouse GARP monoclonal antibody (ALX-804-867-C100, Enzo Life Sciences) was first tested by western blot in untransfected and hGARP-transfected HEK293 cells and by IHC using hGARP-transfected and control vector-transfected mouse Pre-B leukemic cells 70Z/3.

PDA tumor and normal pancreas isolated from KPC mice were fixed in 10% formalin, embedded in paraffin, transferred to slides, and stained by IHC. During antigen retrieval, the EDTA buffer was used for integrin αV antibodies for 30 min in a steamer; and for integrin β8 antibody, the Citrate buffer was used. Slides were then blocked with peroxidase block (Dako, Denmark) for 5 min, followed by avidin and biotin blocking (Vector Labs, CA, USA) for 15 min each at room temperature. Primary antibodies including rabbit anti-mouse integrin αV monoclonal antibody (1:250), and rabbit anti-mouse integrin β8 antibody (1:500) were used in incubation for 1 h followed by biotinylated goat anti-rabbit IgG antibody (1:200, BA-1000, Vector Labs), respectively, for 1 h. Avidin–biotin–peroxidase (ABC) complex (Vectastain ABC kit, PK-4000, Vector Labs) was added for 30 min. DAB hydrochloride (Labs SK-4100, Vector Labs) was used for development.

Multiplex and duplex IHC were performed using the sequential staining and striping method on the mouse and human PDA tissue as previously described.^40,41^ In brief, slides were incubated with primary antibody for 30 min for anti-mouse GARP monoclonal antibody (1:1000, ALX-804-867-C100, Enzo Life Sciences), and mouse anti-human GARP antibody (1: 200, ab194813, Abcam), anti-mouse F4/80 antibody (1:250, 7007, Cell Signaling), anti-human CD68 antibody (1:50, ab783, Abcam), anti-human CD163 antibody (1:100, MA5-11458, Invitrogen), and secondary anti-mouse or anti-rabbit antibodies (Nacalai USA) for 30 min. Lastly, slides were developed with 3-amino-9-ethylcarbazole (AEC, Vector Laboratories), scanned, and stripped to allow for subsequent antibody staining.

### Lucifer yellow dye-coupling assay

Lucifer yellow dye-coupling assay was performed according to the literature.^42^ KPC tumor cells were pre-stained with 5% Lucifer yellow CH solution (Sigma) and then co-cultured with macrophages for 24 h.

### Metabolism assays

Mouse M0, M1, and M2 macrophages were co-cultured with mouse KPC PDA tumor cells for 48 h followed by macrophage isolation using CD11b^+^ EasySep kit (StemCell, Vancouver, Canada). Isolated macrophages were then plated in 96-well plate at a density of 5 × 10^5^ cells/well, respectively, for 20 min in a 37 °C cell culture incubator according to the manufacturer’s instructions. Tetramethylrhodamine Methyl Ester (TMRM) (Abcam) and 2-(N-(7-Nitrobenz-2-oxa-1,3-diazol-4-yl)Amino)−2-deoxyglucose (2-NBDG) (Biovision) fluorescent signal was collected using PE channel using the CytoFLEX flow cytometer (Beckman Coulter).

### RNA preparation and genomic DNA preparation

Total RNA was extracted with the TRIzol reagent (Life Technology) according to the manufacturer’s guide. cDNA was synthesized by ReadyScript^®^ cDNA Synthesis kit (Sigma). Genomic DNA from cells was extracted and converted with DNeasy Blood & Tissue Kit (Qiagen Inc, Valencia, CA, USA) and EZ DNA Methylation-Gold™ Kit (Zymo Research, Irvine, CA, USA) according to the manufacturer’s guideline.

### Quantitative real-time PCR and methylation-specific PCR (MSP)

Quantitative real-time RT-PCR and MSP were performed on StepOnePlus Real-Time PCR System (Thermo Scientific, Hudson, NH, USA). RT-PCR primers used are listed in Supplementary Table 4. Primers and PCR conditions for MSP are described in Supplementary Table 5. For human MSP experiments, in vitro methylated DNA (IVD) was used as positive control; DNA from DNA methyltransferases DNMT1 (−/−) and DNMT3b (−/−) double-knockout cell line (DKO) was used as negative control. The MSP methylation percentage was calculated as: [1/(1 + $2^{\left({\mathrm{CT}}_{{\mathrm{Meth}}\,{\mathrm{value}}}-{\mathrm{CT}}_{{\mathrm{Umeth}}\,{\mathrm{value}}}\right)}$] ×100%.

### DNA methylation array analysis and RNA-sequencing analysis

The Illumina Infinium MethylationEPIC BeadChip was used for DNA methylation array analysis, which was conducted at the Johns Hopkins School of Medicine Microarray Core. DNBseq platform was used for Transcriptome resequencing (RNA-seq) of the six matched monoculture and co-culture samples. The RNA-seq was conducted by The Beijing Genomics Institute (BGI). We mapped clean reads to reference using Bowtie2, and then calculate gene expression level with RSEM. Merged analysis of DNA methylation array and RNA-sequencing results was performed.

### TCGA raw data collection and analysis

Gene expression data of PDACs were retrieved from the TCGA database (https://portal.gdc.cancer.gov/). Gene expression data of normal samples were retrieved from the Genotype-Tissue Expression (GTEx) database (https://gtexportal.org/home/datasets). The TIMER (https://cistrome.shinyapps.io/timer) and GEPIA database (http://gepia.cancer-pku.cn) analysis methods were used to assess the correlation between the gene expression of integrin αV/β8, GARP, and TGFβ−1 and that of immune markers as well as the estimated immune cells infiltration.^43,44^

### Statistics

All statistical analyses were conducted using Graphpad Prism 5.0 (GraphPad Software, La Jolla, CA, USA) and SPSS17.0. Univariate and multivariate analyses were based on log-rank test and Cox proportional hazards regression model. For comparison of individual variables, the Student’s *t* test (normal distribution data), the Mann–Whitney *U* test (non-normal distribution data) and the Fisher’s exact tests were applied appropriately. Data are presented as means ± SEM. Value of *P* < 0.05 was judged to be significant.

## Supplementary information

Supplementary Material

## Data Availability

All data are available in the main text or supplementary materials. Raw data files for the DNA methylation and RNA-sequencing analysis have been deposited in the NCBI Gene Expression Omnibus under accession numbers GSE121656 and GSE122502.
