# Supplementary material for: Pancreatic cancer cells render tumor-associated macrophages metabolically reprogrammed by a GARP and DNA methylation-mediated mechanism
Source: Signal Transduct Target Ther. 2021 Oct 29;6:366. doi: 10.1038/s41392-021-00769-z (PMC8553927; doi:10.1038/s41392-021-00769-z)
Supplement: Supplementary file 1 — Supplementary Material [file 41392_2021_769_MOESM1_ESM.docx]

Supplementary Materials for

**Pancreatic cancer cells render tumor-associated macrophages metabolically reprogrammed by a GARP and DNA methylation-mediated mechanism**

Mengwen Zhang^1,2,^ ^†,‡^ , Xingyi Pan^1,2,3,4, †^, Kenji Fujiwara^1,2,3, §^, Noelle Jurcak^1,2,3,4^, Stephen Muth^1,2,3^, Jiaojiao Zhou^1,2, ‡^, Qian Xiao^1,2, ‡^, Anqi Li^6^, Xu Che^1,2,3, ¶^, Zihai Li^6^, Lei Zheng^1,2,3,4,5,*^

Correspondence to: [lzheng6@jhmi.edu](mailto:lzheng6@jhmi.edu);

**This PDF file includes:**

**Figures. S1 to S7**

**Tables S1 to S4:**

**Figure. S1.**

**
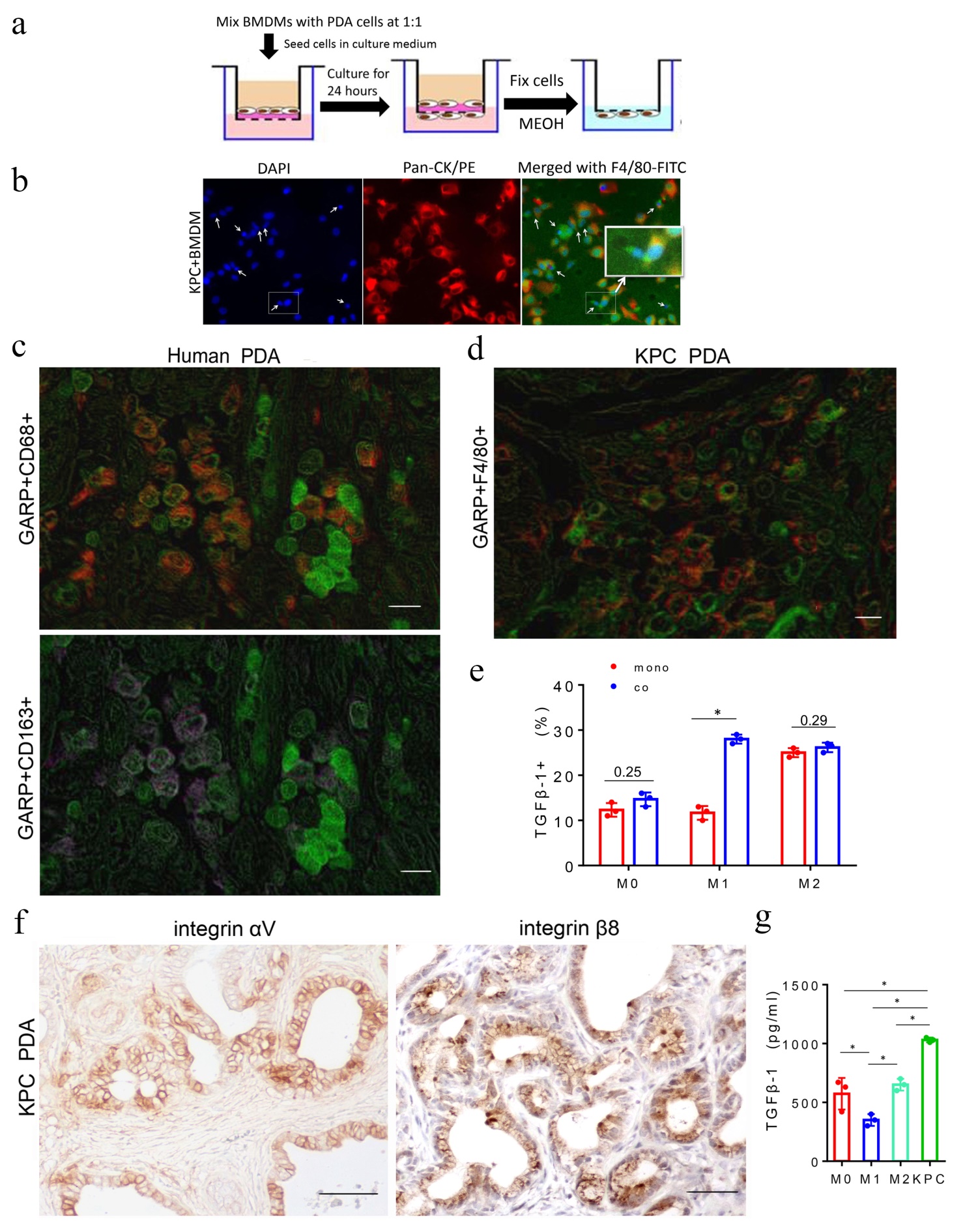
**

**Figure. S1. PDA cells and macrophages co-migrate in the trans-well system. (a)** Scheme of the in vitro tumor-macrophage co-migration assay. KPC tumor cells and mouse BMDMs were mixed in a ratio of 1:1, seeded in the top chamber and allowed to migrate toward the lower chamber for 24 hours**. (b)** Immunofluorescence staining of KPC tumor cells co-migrated with BMDMs onto the coverslips in the lower chambers. KPC tumor cells were stained with Pan-CK-PE; BMDMs were stained with F4/80-FITC; and the nucleus of BMDMs and KPC tumor cells were stained with DAPI. Co-migration of KPC tumor cells and macrophages were indicated by white arrows. **(c)** Enlarged images of **Figure 2e**. Scale bar: 50 μm. Percentages of GARP+CD163-CD68+ M1-like TAMs among M1-like TAMs were 94.3+/-5.4% and percentages of GARP+CD163+CD68+ M2-like TAMs among M2-like TAMs were 76.8+/-4.3% in PDAs tested and quantified (n=15). **(d)** Enlarged images of **Figure 2f.** Scale bar: 50 μm. Percentages of GARP^+^F4/80^+^ macrophages among F4/80+ macrophages were 82.7+/-3.9% in PDAs from KPC mice. **(e)** Cell surface-bound TGFβ-1 on M0, M1-like, M2-like macrophages after co-cultured with KPC tumor cells was measured using flow cytometry. M0, M1-like, and M2-like macrophages were co-cultured with KPC tumor cells for 24 hours. Macrophages were harvested using CD11b+ EasySep kit. Isolated macrophages were then incubated with TGFβ-1-Biotin followed by incubation of Streptavidin-PE. Data are percentages of TGFβ-1 positive cells from technical triplicates and representative of two experiments. *P <0 .05 (Non-parametric Mann-Whitney U test). **(f)** Enlarged images of **Figure 2j**. Scale bar: 100 μm. All PDAs tested are strongly positive for integrin αV and β8. **(g)** TGFβ-1 ELISA was performed with the culture medium of M0, M1-like, M2-like macrophages and KPC cells. Data are means ± SEM from 3 technical replicates and representative of two experiments. *P < 0.05 (One-way ANOVA test adjusted for multiple comparisons).

**Figure. S2.**


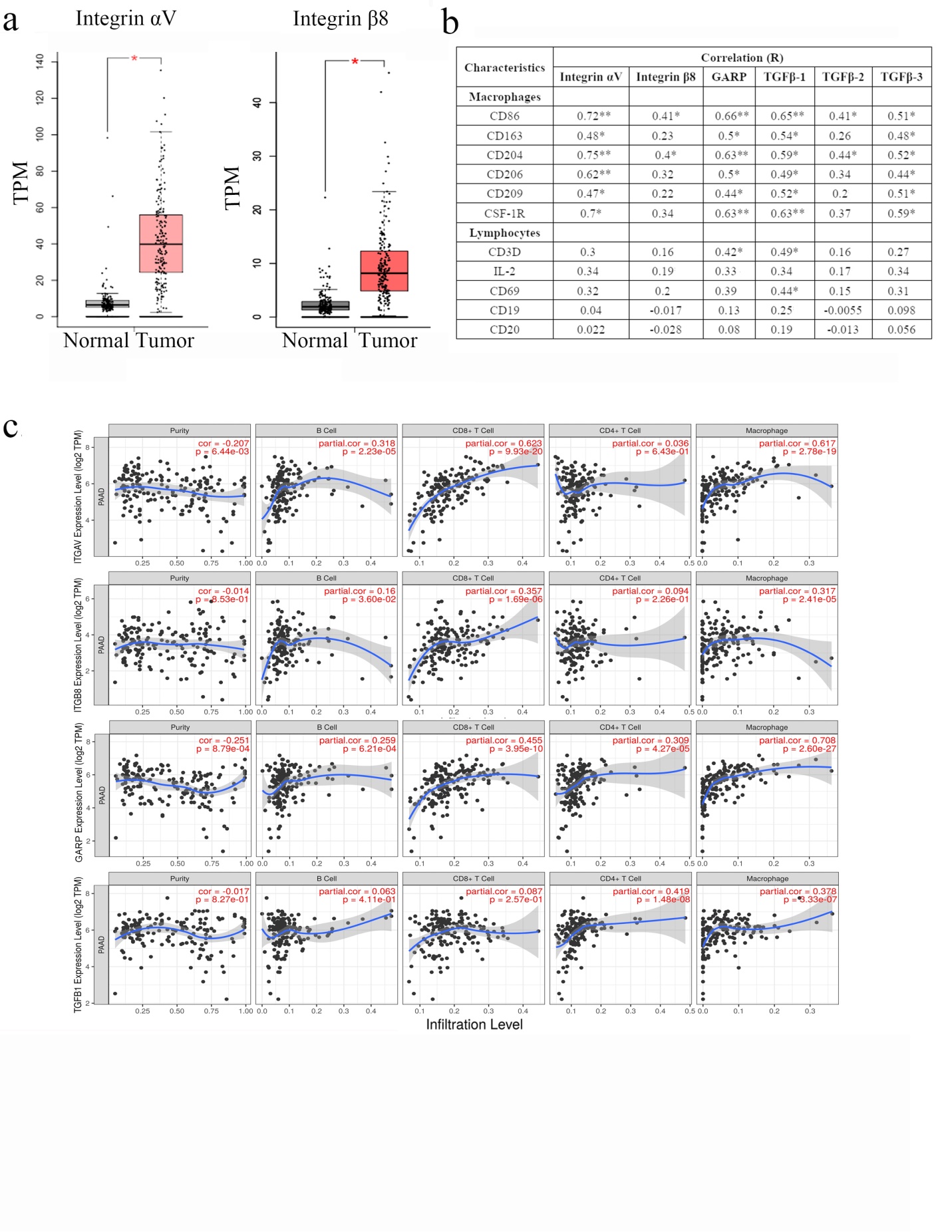


Figure. S2. TCGA data analysis of the gene expression of integrin αV/β8, GARP, and TGF-β in PDA and its correlation with the expression of immune cell marker genes and the estimated infiltration of immune cells. (a) Comparison of the mRNA expression levels of integrin subunit αV and β8 genes, respectively, between human PDAs (n=171) and normal pancreas tissues (n=179) according to the analysis of TGCA and GTEx data. Data was generated from GEPIA ^1^ (http://gepia2.cancer-pku.cn/#general) * p < 0.05 (Wilcoxon rank sum test). (b) GEPIA ^1^ analysis of the correlation between the mRNA expression level of integrin αV/β8, GARP, TGF-β1, TGF-β2, and TGF-β3 and that of immune cell marker genes. ** indicates a Pearson R value between 0.6-0.8, which is considered to be a strong correlation; * indicates a Pearson R value between 0.4-0.6, which is a: moderately strong correlation. >0.8, very strong correlation; 0.2-0.4, weak correlation; <0.2, no correlation. (c) The TIMER ^2^ analysis of the correlation between the mRNA expression level of integrin αV/β8, GARP, TGF-β1 expression and the level of immune cell infiltration estimated by the mRNA expression of the signature genes in human PDAs (https://cistrome.shinyapps.io/timer/). The Spearman’s correlation analysis method was used. The closer the partial correlation (cor) value is to, the stronger the correlation is.

**Figure. S3.**

**
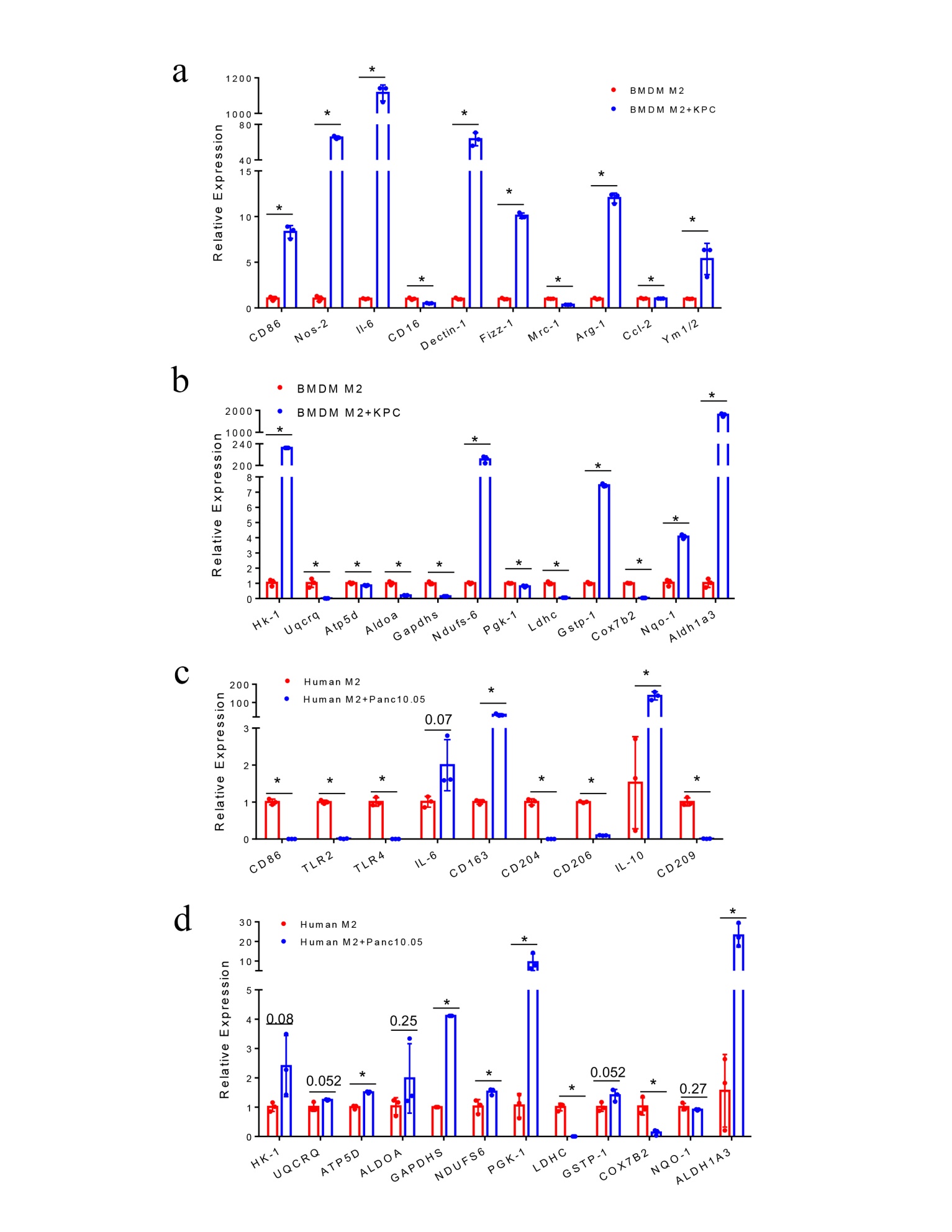
**

**Figure. S3. M2-like macrophages are not metabolically reprogrammed by PDA tumor cells. (a)** Expression of M1 and M2 marker genes was examined in mouse M2-like macrophages after co-culturing with KPC tumor cells. The mRNA expression of these genes was measured by real-time PCR and *β-actin* was used for normalization. Data are means ± SEM from 3 technical replicates.*P <0.05 (Non-parametric Mann-Whitney U test). **(b)** Expression of the key genes in the glucose metabolism and OXPHOS were examined in mouse M2-like macrophages after co-culturing with KPC tumor cells. The mRNA expression of these genes was measured as described above. *P <0.05 (Non-parametric Mann-Whitney U test). **(c)** Expression of M1 and M2 marker genes was examined in human M2-like macrophages after co-culturing with Panc 10.05 tumor cells. The mRNA expression of these genes was measured as described above. *P <0.05 (Non-parametric Mann-Whitney U test). **(d)** The expression of the metabolism genes in human M2-like macrophages was examined after co-culturing with the Panc 10.05 tumor cells. Data are presented as the mean ± SEM from 3 independent biological replicates. All RNA samples were evaluated for RNA integrity and purity. Data are means ± SEM from technical duplicates. *P <0.05 (Non-parametric Mann-Whitney U test). All results are representative of experiments repeated at least twice unless indicated otherwise.

**Figure. S4.**

**
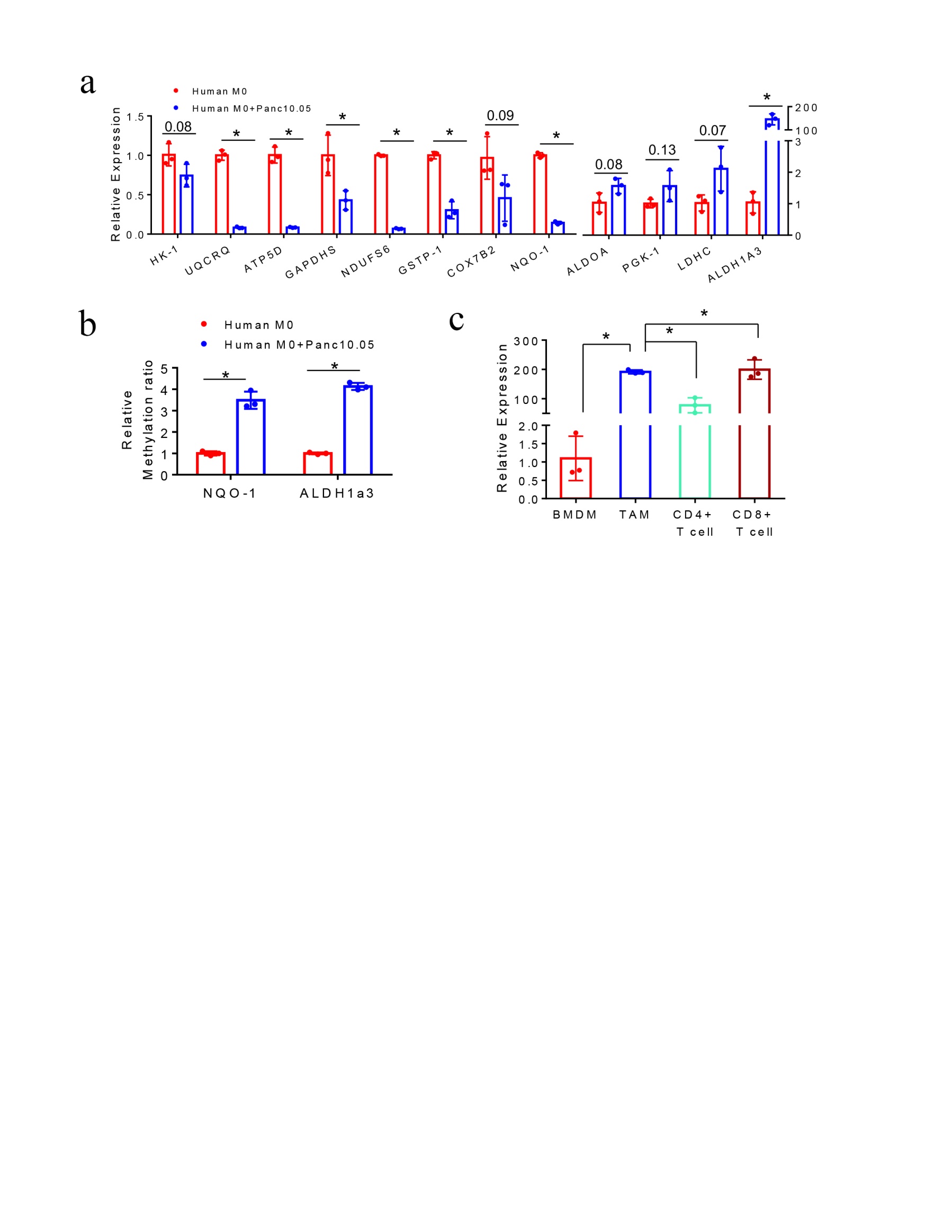
**

**Figure. S4. M0 macrophages can be metabolically reprogrammed by PDA tumor cells. (a)** Expression of metabolic genes was examined in human M0 macrophages after co-culturing with Panc10.05 tumor cells. The mRNA expression of these genes was measured by real-time PCR and β-actin was used for normalization. Data are means ± SEM from 3 technical replicates.*P <0.05 (Non-parametric Mann-Whitney U test). **(b)** Methylation of NQO-1 and ALDH1a3 were examined in human M0 macrophages after co-culturing with Panc10.05 tumor cells. The relative methylation ratio of these genes was measured as described above. *P <0.05 (Two-tailed paired t-test). **(c)** The expression of *Aldh1a3* in pancreatic TAMs, CD4^+^ T cells, CD8^+^ T cells and BMDMs from the same KPC mice was examined. The pancreatic tumor immune cells and BMDMs were from the same mice with Fig. 6a. Data are presented as the mean ± SEM from 3 independent biological replicates. The mRNA expression of these genes was measured by real-time PCR and *β-actin* was used for normalization. Data are means ± SEM from 3 technical replicates. *P <0.05 (Non-parametric Mann-Whitney U test).

**Figure. S5.**


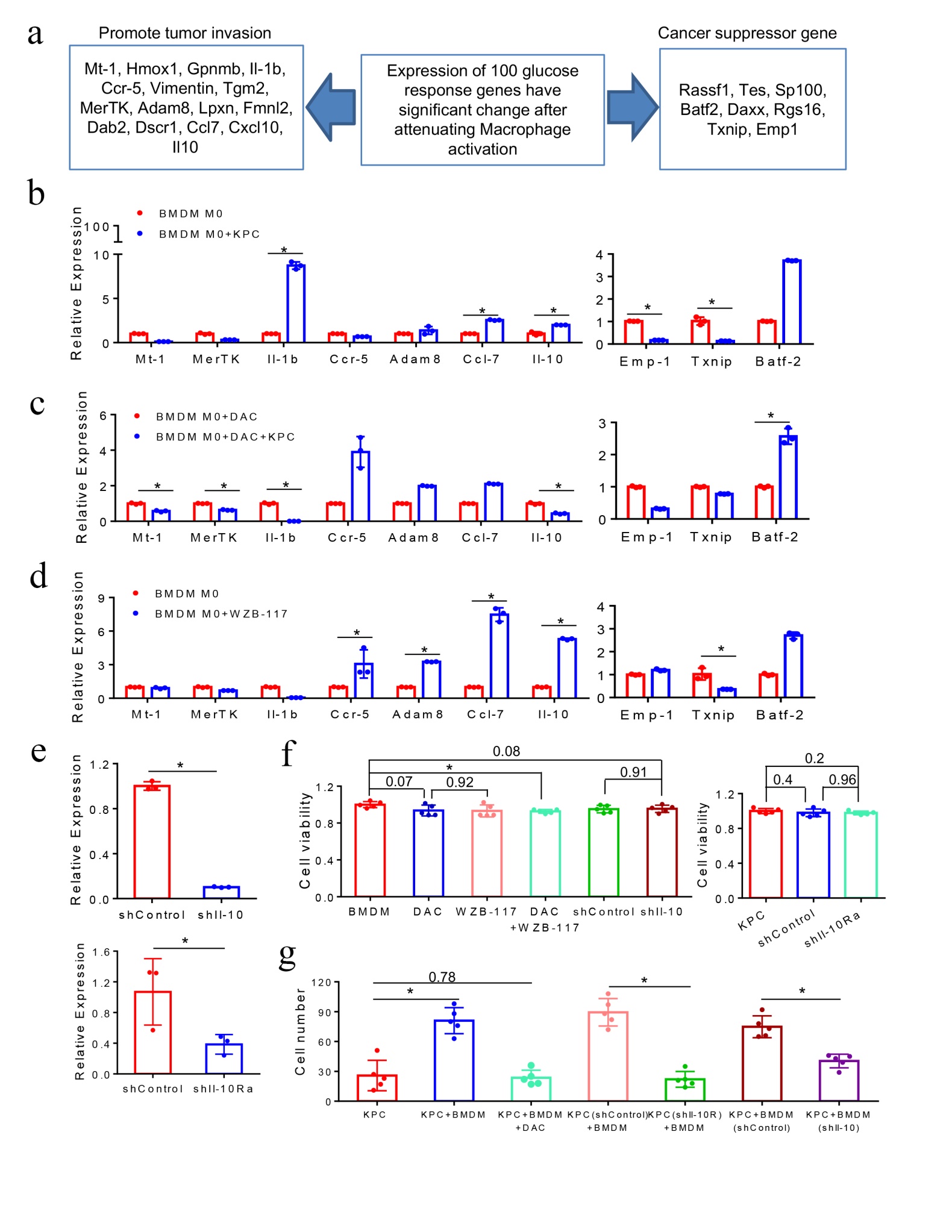


**Figure. S5. Tumor-educated macrophages promote tumor migration through the *Il-10/Il-10* receptor pathway. (a)** Selection of 15 glucose-response genes known to be pro-tumoral and 8 genes known to be anti-tumoral. **(b, c, d)** The expression of 3 anti-tumor genes and 7 pro-tumoral genes whose products are either expressed on the cell surface or secreted were examined in mouse BMDMs or DAC pre-treated BMDMs after co-culturing with KPC tumor cells, or after WZB-117 treatment. *Il-10* mRNA expression in mouse BMDMs was increased after the WZB-117 treatment or co-culture with the KPC tumor cells. DAC reversed the tumor induced *Il-10* overexpression in BMDMs. The mRNA expression of these key genes was measured by real-time PCR and *β-actin* was used for normalization. Data are means ± SEM from 3 technical replicates. *P <0.05 (Non-parametric Mann-Whitney U test). **(e)** *Il-10* and *Il-10R* mRNA expression in mouse BMDMs were knockdown by shIl-10 and shIl-10Ra. The mRNA expression of these key genes was measured by real-time PCR and *β-actin* was used for normalization. Data are means ± SEM from 3 technical replicates. *P <0.05 (Non-parametric Mann-Whitney U test). **(f)** Cell viability and proliferation of BMDMs and KPC cells was evaluated by the CCK-8 assay after incubating with DAC, WZB-117, shControl lentivirus, sh*Il-10* lentivirus or sh*Il-10Ra* lentivirus for 24 hours. *P <0.05 (Non-parametric Mann-Whitney U test). **(g)** Migratory activity of KPC cells co-cultured with mouse BMDMs, DAC pre-treated BMDMs, *Il-10* knock-out BMDMs or *Il-10R* knock-out BMDMs was measured by trans-well assay as described before. The migrated KPC tumor cells were examined by immunofluorescent staining with Pan-CK-FITC antibody. The number of migrated KPC tumor cells was counted from 3 individual visions. Data are means ± SEM from technical duplicates and representative of two experiments. *P <0.05 (One-way ANOVA test adjusted for multiple comparisons). All results are representative of experiments repeated at least twice unless indicated otherwise.

**Figure. S6.**

**
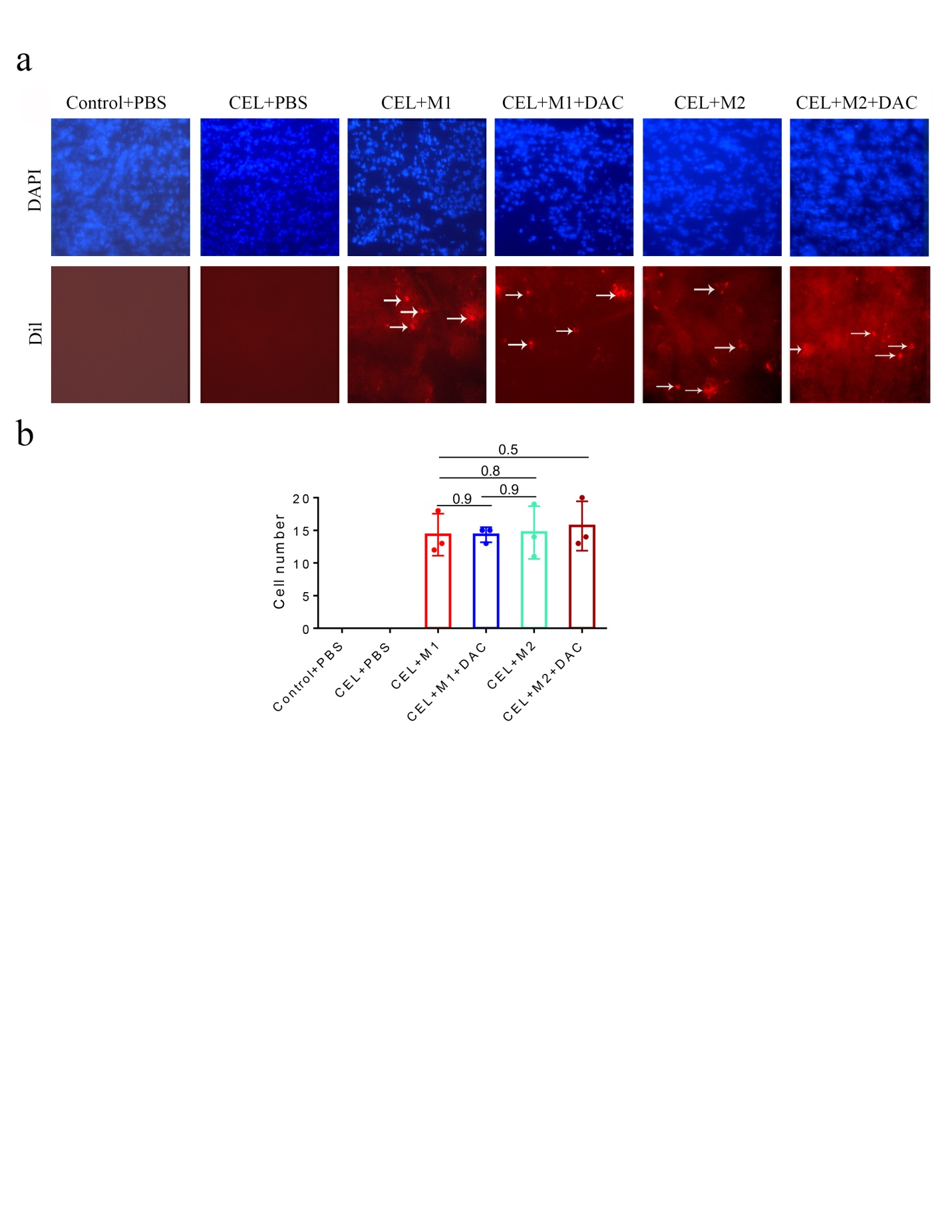
**

**Figure. S6. Exogenous macrophages infiltrate the PDA tumors. (a)** Fluorescence microscope examination of exogenous macrophages pre-labeled by Vybrant Dil in all treatment groups of the experiment in **Figure 5c**. Arrow indicates Vybrant Dil-positive macrophages in pancreas tissues. Scale bar: 50 μm. **(b)** The average cell number counts of exogenous macrophages in one 20x microscopic field. Three fields were randomly chosen from each mouse’s pancreas in every treatment group as indicated. P＞0.05 (One-way ANOVA test adjusted for multiple comparisons between four treatment groups as indicated).

**Figure. S7.**

**
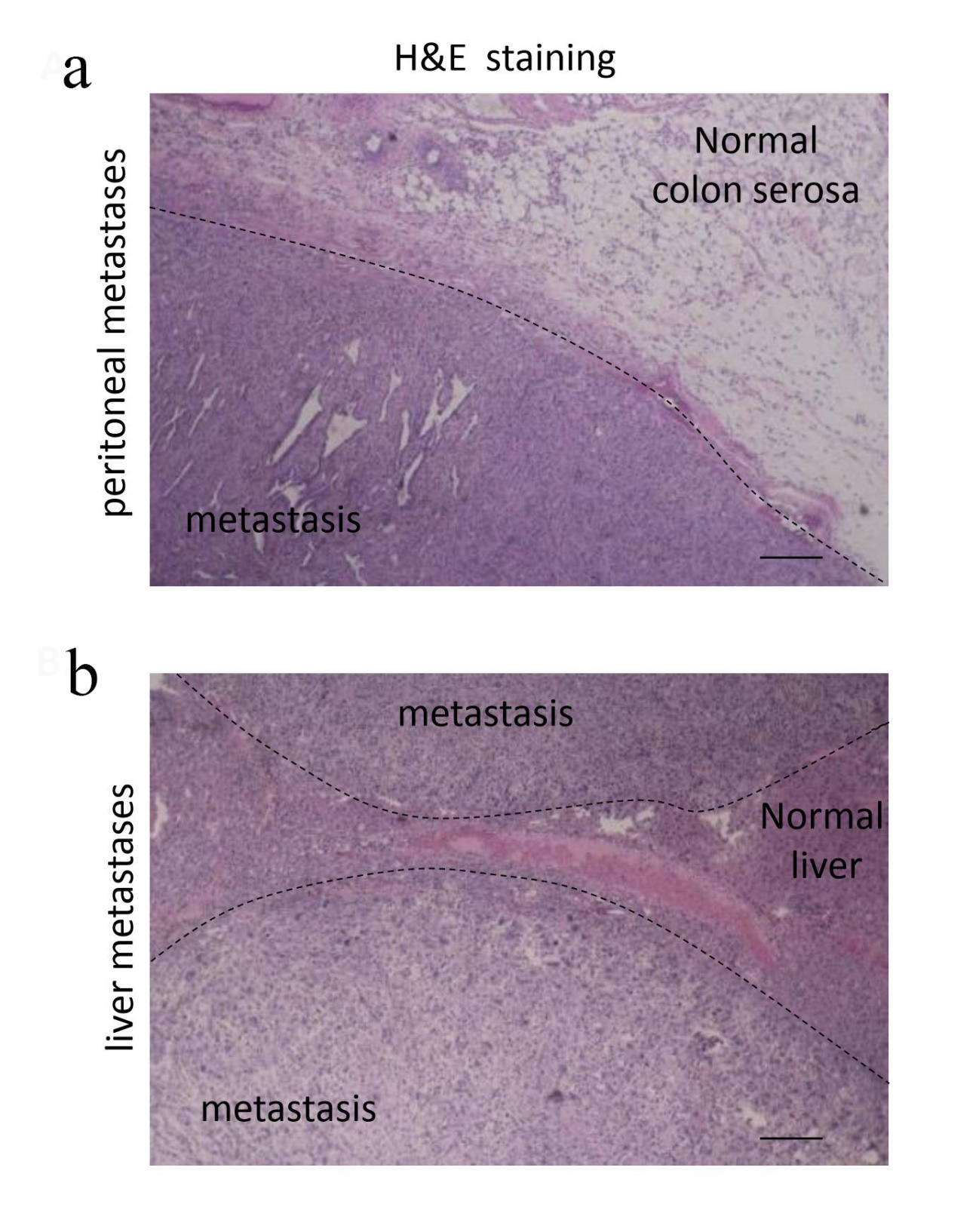
**

**Figure. S7. Representative histopathology images of peritoneal and liver metastases of mouse PDA. (a)** Representative H&E staining of peritoneal metastasis. **(b)** Representative H&E staining of liver metastases from the CEL+M1 group. Magnification: ×40.

**Table S1. Selection of metabolism genes for the current study.**

| **ALDH1a3 related genes (Genes in the Glucose Metabolism Pathway)** | **Genes selected to be validated** |
| --- | --- |
| ALDH3a1; FAHD-1; GSTP-1; ACADM; HK-3;  HK-1; PFKP; ALDOA; GAPDHS; PGK-1; PGAM-1; LDHB; LDHC | ALDH1a3; HK-1; ALDOA; GSTP-1, GAPDHS; PGK-1; LDHC |
| **ALDH1a3 related genes (Genes in other Metabolism Pathways)** | **Genes selected to be validated** |
| TPO; GSTA-4; GSTM-4; GSTK-1;  UGT2A3; CYP3A43; ABAT | None |
| **NQO-1 related genes ( Genes in the OXPHOS )** | **Genes selected to be validated** |
| NDUFS6; NDUFA10; NDUFA12; NDUFB9; UQCRQ; COX7A1; COX7B2; ATP5D; ATP5G2; ATP6V1E1; ATP6V0D1; ATP6V0D2; ATP6V0E1; ATP4A | NQO-1; NDUFS6; UQCRQ; COX7B2; ATP5D; |

**Table S2. Summary of the analysis of the metabolism genes selected for validation with MSP and RT-PCR with M1-like and M2-like macrophages upon co-culturing with PDA tumor cells.**

| **ALDH1a3 related genes (Genes in the Glucose Metabolism Pathway)** |  |  |  | **ALDH1a3** | **HK1** | **ALDOA** | **GAPDHS** | **PGK1** | **LDHC** |
| --- | --- | --- | --- | --- | --- | --- | --- | --- | --- |
|  | **M1 vs. M1.CO** | **Human** | **DNA methylation Array** | **M** | **M** | **M** | **M** | **-** | **M** |
|  |  |  | **MSP** | **M** | **/** | **/** | **/** | **/** | **/** |
|  |  |  | **RNA Sequence** | **↑** | **↑** | **↑** | **/** | **↓** | **↓** |
|  |  |  | **RT-PCR** | **↓** | **↓** | **↓** | **↓** | **↓** | **↓** |
|  |  | **Mouse** | **MSP** | **M** | **/** | **/** | **/** | **/** | **/** |
|  |  |  | **RT-PCR** | **↓** | **↓** | **↓** | **↓** | **↓** | **↓** |
|  | **M2 vs. M2.CO** | **Human** | **DNA methylation Array** | **M** | **M** | **M** | **-** | **-** | **-** |
|  |  |  | **MSP** | **-** | **/** | **/** | **/** | **/** | **/** |
|  |  |  | **RNA Sequence** | **-** | **↑** | **↑** | **/** | **↑** | **/** |
|  |  |  | **RT-PCR** | **-** | **↓** | **↓** | **-** | **↑** | **-** |
|  |  | **Mouse** | **MSP** | **-** | **/** | **/** | **/** | **/** | **/** |
|  |  |  | **RT-PCR** | **↑** | **↑** | **-** | **-** | **-** | **-** |
|  |  |  |  | **NQO-1** | **NDUFS6** | **UQCRQ** | **COX7B2** | **ATP5D** |  |
| **NQO-1 related genes ( Genes in the OXPHOS )** | **M1 vs. M1.CO** | **Human** | **DNA methylation Array** | **-** | **M** | **-** | **M** | **M** |  |
|  |  |  | **MSP** | **M** | **/** | **/** | **/** | **/** |  |
|  |  |  | **RNA Sequence** | **↑** | **↓** | **↓** | **/** | **↑** |  |
|  |  |  | **RT-PCR** | **↓** | **↓** | **↓** | **↓** | **↓** |  |
|  |  | **Mouse** | **MSP** | **M** | **/** | **/** | **/** | **/** |  |
|  |  |  | **RT-PCR** | **↓** | **↓** | **-** | **↓** | **↓** |  |
|  | **M2 vs. M2.CO** | **Human** | **DNA methylation Array** | **-** | **-** | **-** | **-** | **M** |  |
|  |  |  | **MSP** | **-** | **/** | **/** | **/** | **/** |  |
|  |  |  | **RNA Sequence** | **↑** | **↑** | **↑** | **/** | **↑** |  |
|  |  |  | **RT-PCR** | **-** | **-** | **-** | **-** | **↓** |  |
|  |  | **Mouse** | **MSP** | **-** | **/** | **/** | **/** | **/** |  |
|  |  |  | **RT-PCR** | **↑** | **↑** | **↑** | **-** | **-** |  |

**Table S3. PCR Primers.**

| **Mouse metabolism genes** | | Forward primer (5'-3') | Reverse primer (5'-3') |
| --- | --- | --- | --- |
| *Gstp-1* |  | ATGCCACCATACACCATTGTC | GGGAGCTGCCCATACAGAC |
| *Hk-1* | NM_001146100 | GAGTCTGAGGTCTACGACACC | CCCACGGGTAATTTCTTGTCC |
| *Aldoa* | [NM_001177308](http://www.ncbi.nlm.nih.gov/entrez/query.fcgi?cmd=Search&db=Nucleotide&term=NM_001177308) | AGTCCACCGGAAGCATTGC | CAGCCCCTGGGTAGTTGTC |
| *Gapdhs* | [NM_008085](http://www.ncbi.nlm.nih.gov/entrez/query.fcgi?cmd=Search&db=Nucleotide&term=NM_008085) | TGGATTTGGACGCATTGGTC | TTTGCACTGGTACGTGTTGAT |
| *Pgk-1* | [NM_008828](http://www.ncbi.nlm.nih.gov/entrez/query.fcgi?cmd=Search&db=Nucleotide&term=NM_008828) | ATGTCGCTTTCCAACAAGCTG | GCTCCATTGTCCAAGCAGAAT |
| *Ldhc* | [NM_013580](http://www.ncbi.nlm.nih.gov/entrez/query.fcgi?cmd=Search&db=Nucleotide&term=NM_013580) | TTGACGCTGATACGAACAAACT | AGACGATTTTTGGAGTGCTAAGG |
| *Ndufs-6* | [NM_010888](http://www.ncbi.nlm.nih.gov/entrez/query.fcgi?cmd=Search&db=Nucleotide&term=NM_010888) | GGGGAAAAGATCACGCATACC | CAAAACGAACCCTCCTGTAGTC |
| *Uqcrq* | [NM_025352](http://www.ncbi.nlm.nih.gov/entrez/query.fcgi?cmd=Search&db=Nucleotide&term=NM_025352) | CCTACAGCTTGTCGCCCTTT | GATCAGGTAGACCACTACAAACG |
| *Cox7b2* | [NP_084328](http://www.ncbi.nlm.nih.gov/entrez/viewer.fcgi?val=NP_084328) | TCTCAAGACTCCAAGCATTCTGA | GACAGGTTCCACTCCACACC |
| *Atp5d* | [NM_025313](http://www.ncbi.nlm.nih.gov/entrez/query.fcgi?cmd=Search&db=Nucleotide&term=NM_025313) | CCACACTACAGGTCCTACGG | CACAGAGGAGTCGGCATTCA |
| *Aldh1a3* | NM_053080 | GGGTCACACTGGAGCTAGGA | CTGGCCTCTTCTTGGCGAA |
| *Nqo-1* |  | AGGATGGGAGGTACTCGAA | AGGCGTCCTTCCTTATATGC |
| *β-actin* | [NM_007393](http://www.ncbi.nlm.nih.gov/entrez/query.fcgi?cmd=Search&db=Nucleotide&term=NM_007393) | ATGCTCCCCGGGCTGTAT | ATAGGAGTCCTTCTGACCCATTC |
| **Mouse M1 marker** | |  |  |
| *CD16* | [NM_010188](http://www.ncbi.nlm.nih.gov/entrez/query.fcgi?cmd=Search&db=Nucleotide&term=NM_010188) | TTTGGACACCCAGATGTTTCAG | GTCTTCCTTGAGCACCTGGATC |
| *CD32* | [NM_001077189](http://www.ncbi.nlm.nih.gov/entrez/query.fcgi?cmd=Search&db=Nucleotide&term=NM_001077189) | AATCCTGCCGTTCCTACTGATC | GTGTCACCGTGTCTTCCTTGAG |
| *CD86* | [NM_019388](http://www.ncbi.nlm.nih.gov/entrez/query.fcgi?cmd=Search&db=Nucleotide&term=NM_019388) | TTGTGTGTGTTCTGGAAACGGAG | AACTTAGAGGCTGTGTTGCTGGG |
| *Nos2* | [NM_010927](http://www.ncbi.nlm.nih.gov/entrez/query.fcgi?cmd=Search&db=Nucleotide&term=NM_010927) | CCCTTCAATGGTTGGTACATGG | ACATTGATCTCCGTGACAGCC |
| *Il-6* | [NM_031168](http://www.ncbi.nlm.nih.gov/entrez/query.fcgi?cmd=Search&db=Nucleotide&term=NM_031168) | ATTTCCAATGCTCTCCTAACAG | TGTCCACAAACTGATATGCTTAG |
| **Mouse M2 marker** | | Forward primer (5'-3') | Reverse primer (5'-3') |
| *Arg1* | [NM_007482](http://www.ncbi.nlm.nih.gov/entrez/query.fcgi?cmd=Search&db=Nucleotide&term=NM_007482) | GAACACGGCAGTGGCTTTAAC | TGCTTAGCTCTGTCTGCTTTGC |
| *Ym1/2* |  | AGAAGGGAGTTTCAAACCTGGT | GTCTTGCTCATGTGTGTAAGTGA |
| *Ccl-2* | NM_011333 | TGCATCTGCCCTAAGGTCTTCA | GTGCTTGAGGTGGTTGTGGAAA |
| *Mrc-1* |  | CTCTGTTCAGCTATTGGACGC | TGGCACTCCCAAACATAATTTGA |
| *dectin-1* |  | GACTTCAGCACTCAAGACATCC | TTGTGTCGCCAAAATGCTAGG |
| *Fizz1* |  | CCAATCCAGCTAACTATCCCTCC | CCAGTCAACGAGTAAGCACAG |
| **Mouse invasion genes** | | Forward primer (5'-3') | Reverse primer (5'-3') |
| *Mt-1* | BC027262 | AAGAGTGAGTTGGGACACCTT | CGAGACAATACAATGGCCTCC |
| *Il-1b* | NM_008361 | TTCAGGCAGGCAGTATCACTC | GAAGGTCCACGGGAAAGACAC |
| *Ccr-5* | NM_009917 | ATGGATTTTCAAGGGTCAGTTCC | CTGAGCCGCAATTTGTTTCAC |
| *Adam8* | NM_007403 | GCAGGACCATTGCCTCTACC | TGGACCCAACTCGGAAAAAGC |
| *Ccl7* | NM_013654 | CCACATGCTGCTATGTCAAGA | ACACCGACTACTGGTGATCCT |
| *Cxcl10* | NM_021274 | CCAAGTGCTGCCGTCATTTTC | GGCTCGCAGGGATGATTTCAA |
| *Il-10* | NM_010548 | CTTACTGACTGGCATGAGGATCA | GCAGCTCTAGGAGCATGTGG |
| *Txnip* | NM_001009935 | GGCCGGACGGGTAATAGTG | AGCGCAAGTAGTCCAAAGTCT |
| *Emp1* | NM_010128 | TTGGTGCTACTGGCTGGTCT | AGCATCTTCATTGCCGTAGGA |
| *MerTK* | NM_008587 | CTCCTGAGCCCGTCAATATCT | AGACCAGGTACGGTTAGGACA |
| *Batf2* | NM_028967 | GAAGCACACCAGTAAGGCG | GCACAGGCGTTCATGCAAG |
| **Human metabolism genes** | | Forward primer (5'-3') | Reverse primer (5'-3') |
| *GSTP-1* | NM_000852.3 | TTGGGCTCTATGGGAAGGAC | GGGAGATGTATTTGCAGCGGA |
| *HK-1* | [NM_033498](http://www.ncbi.nlm.nih.gov/entrez/query.fcgi?cmd=Search&db=Nucleotide&term=NM_033498) | GCTCTCCGATGAAACTCTCATAG | GGACCTTACGAATGTTGGCAA |
| *ALDOA* | [NM_000034](http://www.ncbi.nlm.nih.gov/entrez/query.fcgi?cmd=Search&db=Nucleotide&term=NM_000034) | ATGCCCTACCAATATCCAGCA | GCTCCCAGTGGACTCATCTG |
| *GAPDHS* | [NM_014364](http://www.ncbi.nlm.nih.gov/entrez/query.fcgi?cmd=Search&db=Nucleotide&term=NM_014364) | CTCACCGGATGCACCAATGTT | CGCGTTGCTCACAATGTTCAT |
| *PGK-1* | [NM_000291](http://www.ncbi.nlm.nih.gov/entrez/query.fcgi?cmd=Search&db=Nucleotide&term=NM_000291) | TGGACGTTAAAGGGAAGCGG | GCTCATAAGGACTACCGACTTGG |
| *LDHC* | [NM_017448](http://www.ncbi.nlm.nih.gov/entrez/query.fcgi?cmd=Search&db=Nucleotide&term=NM_017448) | AGAACATGGTGATTCTAGTGTGC | ACAGTCCAATAGCCCAAGAGG |
| *NDUFS-6* | [NM_004553](http://www.ncbi.nlm.nih.gov/entrez/query.fcgi?cmd=Search&db=Nucleotide&term=NM_004553) | TTCGGTTTGTAGGTCGTCAGA | CCATCGCACGCTATCACCC |
| *UQCRQ* | [NM_014402](http://www.ncbi.nlm.nih.gov/entrez/query.fcgi?cmd=Search&db=Nucleotide&term=NM_014402) | CGCGAGTTTGGGAATCTGAC | TAGTGAAGACGTGCGGATAGG |
| *COX7B2* | [NM_130902](http://www.ncbi.nlm.nih.gov/entrez/query.fcgi?cmd=Search&db=Nucleotide&term=NM_130902) | CCAGAAATGCACTAAGCAGTCT | ACCCATGTAGCAACACAGAAAG |
| *ATP5D* | [NM_001001975](http://www.ncbi.nlm.nih.gov/entrez/query.fcgi?cmd=Search&db=Nucleotide&term=NM_001001975) | TCCCACGCAGGTGTTCTTC | GGAACCGCTGCTCACAAAGT |
| *ALDH1a3* | [NM_000693](http://www.ncbi.nlm.nih.gov/entrez/query.fcgi?cmd=Search&db=Nucleotide&term=NM_000693) | ACCTCTCACCGCCCTTTATCT | GTGAAGGCGATCTTGTTGATCT |
| *NQO-1* | [NM_001025433](http://www.ncbi.nlm.nih.gov/entrez/query.fcgi?cmd=Search&db=Nucleotide&term=NM_001025433) | GAAGAGCACTGATCGTACTGGC | GGATACTGAAAGTTCGCAGGG |
| *β-ACTIN* | [NM_001101](http://www.ncbi.nlm.nih.gov/entrez/query.fcgi?cmd=Search&db=Nucleotide&term=NM_001101) | CATGTACGTTGCTATCCAGGC | CTCCTTAATGTCACGCACGAT |
| **Human M1 marker** | | Forward primer (5'-3') | Reverse primer (5'-3') |
| *CD86* | [NM_175862](http://www.ncbi.nlm.nih.gov/entrez/query.fcgi?cmd=Search&db=Nucleotide&term=NM_175862) | CTGCTCATCTATACACGGTTACC | GGAAACGTCGTACAGTTCTGTG |
| *TLR2* | [NM_003264](http://www.ncbi.nlm.nih.gov/entrez/query.fcgi?cmd=Search&db=Nucleotide&term=NM_003264) | ATCCTCCAATCAGGCTTCTCT | GGACAGGTCAAGGCTTTTTACA |
| *TLR4* | [NM_138557](http://www.ncbi.nlm.nih.gov/entrez/query.fcgi?cmd=Search&db=Nucleotide&term=NM_138557) | AGACCTGTCCCTGAACCCTAT | CGATGGACTTCTAAACCAGCCA |
| *CXCL10* | [NM_001565](http://www.ncbi.nlm.nih.gov/entrez/query.fcgi?cmd=Search&db=Nucleotide&term=NM_001565) | GTGGCATTCAAGGAGTACCTC | TGATGGCCTTCGATTCTGGATT |
| *IL-6* | [NM_000600](http://www.ncbi.nlm.nih.gov/entrez/query.fcgi?cmd=Search&db=Nucleotide&term=NM_000600) | ACTCACCTCTTCAGAACGAATTG | CCATCTTTGGAAGGTTCAGGTTG |
| *MARCO* | [NM_006770](http://www.ncbi.nlm.nih.gov/entrez/query.fcgi?cmd=Search&db=Nucleotide&term=NM_006770) | CAGCGGGTAGACAACTTCACT | TTGCTCCATCTCGTCCCATAG |
| **Human M2 marker** | | Forward primer (5'-3') | Reverse primer (5'-3') |
| *CD163* | NM_004244 | TTTGTCAACTTGAGTCCCTTCAC | TCCCGCTACACTTGTTTTCAC |
| *CD204* | [NM_002445](http://www.ncbi.nlm.nih.gov/entrez/query.fcgi?cmd=Search&db=Nucleotide&term=NM_002445) | GCAGTGGGATCACTTTCACAA | AGCTGTCATTGAGCGAGCATC |
| *CD206* | [NM_002438](http://www.ncbi.nlm.nih.gov/entrez/query.fcgi?cmd=Search&db=Nucleotide&term=NM_002438) | GGGTTGCTATCACTCTCTATGC | TTTCTTGTCTGTTGCCGTAGTT |
| *IL-10* | [NM_000572](http://www.ncbi.nlm.nih.gov/entrez/query.fcgi?cmd=Search&db=Nucleotide&term=NM_000572) | GACTTTAAGGGTTACCTGGGTTG | TCACATGCGCCTTGATGTCTG |
| *CD209* | [NM_021155](http://www.ncbi.nlm.nih.gov/entrez/query.fcgi?cmd=Search&db=Nucleotide&term=NM_021155) | AATGGCTGGAACGACGACAAA | CAGGAGGCTGCGGACTTTTT |

**Table S4. MSP Primers.**

| **Mouse MSP primer** | |  | Annealing Tm | Cycle |
| --- | --- | --- | --- | --- |
| Aldh1a3-M | Forward | 5’-TTGTTATTGCGATCGTTACGAG-3’ | 54 | 40 |
| Aldh1a3-M | Reverse | 5’-TCTATCTACGCATCGCTAAACG -3’ |  |  |
| Aldh1a3-U | Forward | 5’-GGTTTGTTATTGTGATTGTTATGAG -3’ | 52 | 40 |
| Aldh1a3-U | Reverse | 5’-ACTCTATCTACACATCACTAAACA -3’ |  |  |
| Nqo-1-M | Forward | 5’-TGGAGTTTAGTTTCGTTTTCGTTG -3’ | 56 | 40 |
| Nqo-1-M | Reverse | 5’- GCAATCGTAATACCGAACGCTAAT -3’ |  |  |
| Nqo-1-U | Forward | 5’-TGGAGTTTAGTTTTGTTTTTGTTG -3’ | 56 | 40 |
| Nqo-1-U | Reverse | 5’- GCAATCATAATACCAAACACTAAT -3’ |  |  |
| **Human MSP primer** | |  |  |  |
| NQO1-M | Forward | 5'-AGTTTCGGTTAGGGTCGTTC-3' | 50 | 43 |
| NQO1-M | Reverse | 5'-CCAATACTCGAAAAACGACCG-3' |  |  |
| NQO1-U | Forward | 5'-AGTTTTGGTTAGGGTTGTTTT-3' | 47 | 43 |
| NQO1-U | Reverse | 5'-CCAATACTCAAAAAACAACCA-3' |  |  |
| ALDH1A3-M | Forward | 5'-TCGGTTTCGTAGTTAATTAGGC-3' | 55 | 34 |
| ALDH1A3-M | Reverse | 5'-GACTCGACCCGAACACTACGCA-3' |  |  |
| ALDH1A3-U | Forward | 5'-TTGGTTTTGTAGTTAATTAGGT-3' | 49 | 34 |
| ALDH1A3-U | Reverse | 5'-CAACTCAACCCAAACACTACACA-3' |  |  |

**References in Supplementary Materials**

1. Tang, Z. *et al.*, GEPIA: a web server for cancer and normal gene expression profiling and interactive analyses. *NUCLEIC ACIDS RES* **45** W98 (2017).
2. Li, T. *et al.*, TIMER2.0 for analysis of tumor-infiltrating immune cells. *NUCLEIC ACIDS RES* **48** W509 (2020).
